# Comparison of CRISPR-MAD7 and CRISPR-Cas9 for Gene Disruptions in *Komagataella phaffii*

**DOI:** 10.3390/jof10030197

**Published:** 2024-03-05

**Authors:** Kirill Smirnov, Florian Weiss, Anna-Maria Hatzl, Lukas Rieder, Kjeld Olesen, Sanne Jensen, Anton Glieder

**Affiliations:** 1 Christian Doppler Laboratory for Innovative Pichia pastoris Host and Vector Systems, Institute of Molecular Biotechnology, Graz University of Technology, 8010 Graz, Austria; florian.weiss@tugraz.at (F.W.);; 2 Novo Nordisk A/S, Novo Nordisk Park, DK 2760 Måløv, Denmark

**Keywords:** MAD7 nuclease, CRISPR-Cas9, *Komagataella phaffii*, *Pichia pastoris*, genome editing, efficiency

## Abstract

CRISPR (clustered regularly interspaced short palindromic repeats)-based technologies are powerful, programmable tools for site-directed genome modifications. After successful adaptation and efficient use of CRISPR-Cas9 for genome engineering in methylotrophic yeast *Komagataella phaffii*, a broader variety of employable endonucleases was desired to increase the experimental flexibility and to provide alternatives in case there are specific legal restrictions in industrial research due to the intellectual property rights (IPRs) of third parties. MAD7, an engineered Class 2 Type V Cas nuclease, was promoted as a royalty-free alternative for academic and industrial research and developed by Inscripta (Pleasanton, CA, USA). In this study, for the first time, CRISPR-MAD7 was used for genome editing in *K. phaffii* with a high gene-editing rate (up to 90%), as demonstrated for the three targeted genes coding for glycerol kinase 1 (*GUT1*), red fluorescence protein (*DsRed*), and zeocin resistance gene (Sh ble). Additionally, the genome-editing efficiencies of the CRISPR-MAD7 and CRISPR-Cas9 systems were systematically compared by targeting 259 kinase genes in *K. phaffii*. In this broad testing, the CRISPR-Cas9 had a higher genome-editing rate of about 65%, in comparison to the applied CRISPR-MAD7 toolbox (about 23%).

## 1. Introduction

In the 1970s, Philips Petroleum Company introduced the methylotrophic yeast *Pichia pastoris* for the industrial production of biomass and single-cell protein [1,2]. In the 1980s, *P. pastoris* was evolved as a chassis for heterologous gene expression [1,3,4]. Today, *P. pastoris*, which was deposited by Philips Petroleum Company as the NRRL Y-11430 strain, has been reclassified as *Komagataella phaffii* (*K. phaffii*) [5,6,7], and has become one of the most commonly used organisms for the production of recombinant proteins [1,2,8]. Recent genome sequencing data indicated that *K. phaffii* NRRL Y-11430 most likely is a variant derived from a strain which had been originally deposited by Herman Phaff in 1954 after isolation from exudate flux of a black oak in California [6,7]. The later rise of *K. phaffii* to a leading expression host in industrial applications was caused by a combination of factors: (i) high titers of secreted recombinant protein; (ii) hardly any secreted endogenous proteins simplifying downstream processing; (iii) typical eukaryotic post-translational modifications (PTMs) can be made; and (iv) high cell densities (>100 g/L dry cell weight) are reached while employing affordable media components. Nevertheless, there is still room to improve the *K. phaffii* platform for recombinant protein production [9].

One of the strategies to improve the production of recombinant proteins with respect to yield and PTMs is to specifically tailor the characteristics of already existing strains to the desired application. This strain engineering process is often completed using genome reduction [10]. Genome reduction can be described as a process of removing (physical or functional) cellular genes with the aim that the analysis of the deletion strains reveals genes that are important for metabolic pathways, and, finally, the recombinant protein production [11]. Classically, the integration of knock-out cassettes was used for genome modification in *Saccharomyces cerevisiae* and other yeasts [12]. However, obtaining specially targeted genome modifications in *K. phaffii* using this method has proven to be challenging. This is due to the naturally low homologous recombination (HR) efficiency and the preference of *K. phaffii* for the less specific and indel-prone non-homologous end joining (NHEJ) as the double-strand break (DSB) repair mechanism [13]. Even though the deletion of the *ku70* homolog, a major player in NHEJ, increased the HR efficiency significantly, the knockout elevated susceptibility to DNA damage and resulted in *K. phaffii* phenotypes with reduced growth capacity [14]. Another alternative to this classical approach is to use nuclease-based systems, such as CRISPR-Cas9 for gene disruption in *K. phaffii* [15,16].

These days, a variety of CRISPR-based molecular tools for genome editing are known to the science community. The two most commonly used systems are CRISPR-Cas9 and CRISPR-Cas12a [17,18]. The CRISPR-Cas9 system (Table 1) is typically formed from a type II nuclease Cas9 from *Streptococcus pyogenes* (*Sp*Cas9) and a single guide RNA chimera (sgRNA), which is a fusion of the gRNA (crRNA) and the gRNA scaffold (tracrRNA). In principle, the CRISPR-Cas9 system can be used to introduce DSB at any desired genomic locus, as the *Sp*Cas9 can be directed by altering the gRNA sequence (spacer) of the sgRNA [19]. As a result of the DNA DSB, the cell’s endogenous machinery is triggered, and it repairs the DSB via NHEJ, microhomology-mediated end joining (MMEJ), single-stranded annealing (SSA), or homology directed repairing (HDR), allowing for the introduction of various genomic modifications. The first three repair mechanisms are prone to insertions and deletions (indels), which may cause reading-frame shifts and consequently inactivation of the targeted gene(s). The selection of the repair pathway depends on the configuration of the DNA ends. For instance, NHEJ occurs mainly at blunt end, while short and extensive ends at the break site favor MMEJ and SSA, respectively. HDR allows integration of donor cassettes with homologous overhangs [14,15,20,21,22].

In 2017, information about an alternative to the CRISPR-Cas9 system exploiting a codon-optimized Cas12a nuclease from *Eubacterium rectale* called MAD7 (Table 1) was released by Inscripta (CA, USA). The nuclease still shows 76% nucleotide sequence identity to the native sequence, and in recent years, this MAD7 nuclease was promoted by Inscripta as a royalty-free alternative to Cas9 for most commercial and academic research and development [23]. MAD7 displays a structural similarity to CRISPR-Cas12a (also known as Cpf1) from *Acidaminococcus* species with which the MAD7 shares an amino acid sequence identity of 31% [24]. The CRISPR-MAD7 system had been reported to be functional in a broad spectrum of prokaryotic and eucaryotic organisms like *Bacillus subtilis*, zebrafish, rodents, plant and human cells, as well as filamentous fungi [25,26,27,28,29].

**Table 1:** The main features of CRISPR-Cas9 and CRISPR-MAD7 systems.

Feature	CRISPR-Cas9	CRISPR-MAD7
The source of the nuclease	*Streptococcus pyogenes*	*Eubacterium rectale*
Class CRISPR nuclease	Class 2 nuclease (single, multidomain protein)	Class 2 nuclease (single, multidomain protein)
Type CRISPR nuclease	Type II	Type V
PAM recognition site	5′-NGG-3′ [17]	5′-YTTN-3′ [25]
Cleavage site	3 bp upstream of the PAM site [30]	19 bases downstream of the PAM site on the sense strand, and 23 bases after the complementary PAM site of the anti-sense strand
Cleavage pattern	Blunt cutting [30]	Staggered cutting [31]
sgRNA conformation	One component (but a two-component in native system) [32]	One component

Already, in 2016, Weninger et al. established a highly efficient CRISPR-Cas9 system for *K. phaffii* by testing various combinations of CRISPR-Cas9 components with previously established plasmid and expression systems [15]. The most efficient CRISPR-Cas9 system was obtained by combining a human codon-optimized Cas9 nuclease carrying the nuclear localization signal from Simian Virus 40 large T antigen and a ribozyme-flanked sgRNA, both under the control of the bidirectional, constitutive RNA polymerase II promotor *HTX1* (P*_HTX1_*). The bidirectional expression cassette was cloned in an autonomously replicating plasmid, allowing for the transient expression of the genome-editing tool, in the transformed yeast population due to plasmid loss, caused by the low stability of ARS-based plasmids in *K. phaffii* [33]. The resulting CRISPR-Cas9 system showed a gene-editing efficiency of up to 95% in *K. phaffii* [15,16]. Recently, Dalvie et al. demonstrated the RNA Pol-III mediated expression of sgRNA and reported a simplified, multiplexed cassette for the expression of sgRNA in *K. phaffii* [16]. Moreover, the CRISPR-Cpf1 system was examined in *K. phaffii*, and high editing efficiency was reported [34]. These studies illustrate that there are still a lot of opportunities to expand the CRISPR-based gene-editing tools for *K. phaffii*. To expand the toolbox for genome engineering in *K. phaffii*, we turned our attention to the CRISPR-MAD7 system and investigated the nucleases for the generation of short insertions or deletions (indels) in order to interrupt open coding regions at different positions than with CRISPR-Cas9. To ensure the best functionality, again, we tested several codon optimizations for the MAD7 and multiple combinations of nuclease, nuclear localization signal (NLS), and gRNA scaffold sequences. For initial testing, we targeted the glycerol kinase 1 gene (*GUT1*) locus, as this locus is readily accessible, and gene disruption is easily monitored by decreased cell growth on glycerol as the sole carbon source [15,16,35]. Once functional, we used the established CRISPR-MAD7 system to successfully inactivate various genes located in the genome of the yeast. Finally, a systematic comparison of the established CRISPR-MAD7 system with the existing CRISPR-Cas9 system was performed by assessing the genome-wide-editing efficiencies of both systems for 259 kinases found in the genome of *K. phaffii*.

## 2. Materials and Methods

### 2.1. Chemicals

Single-stranded DNA oligonucleotides were obtained from Integrated DNA Technologies (Leuven, Belgium), genes, and DNA fragments from Twist Bioscience (San Francisco, CA, USA). Restriction enzymes were ordered from Thermo Fisher Scientific (Vienna, Austria) and New England Biolabs (Frankfurt, Germany). For PCR reactions, we used the Q5 Polymerase from New England Biolabs (Frankfurt, Germany). D(+)-biotin was purchased from Sigma-Aldrich (Vienna, Austria), Zeocin™ from InvivoGen (Toulouse, France), and Hygromycin B from Formedium Ltd. (Hunstaton, UK). Difco yeast nitrogen base without amino acids and Bacto yeast extract were obtained from Becton Dickinson (Schwechat, Austria). All other chemicals were purchased from Carl Roth (Karlsruhe, Germany).

### 2.2. Strains

For cloning and plasmid propagation, the *Escherichia coli* Xl1-blue (*E. coli*) strain from the department’s culture collection was used. Competent cell preparation and transformation were performed with and according to the “Mix & Go *E. coli* Transformation Kit & Buffer Set” by Zymo Research (Freiburg, Germany). If not stated otherwise, the *K. phaffii* BSYBG10, a wildtype-like Mut^+^ strain derived from *K. phaffii* NRRL Y-11430 through curing killer-plasmids (bisy GmbH, Hofstätten an der Raab, Austria, previously made by BioGrammatics Inc., Carlsbad, CA, USA. and also distributed as *K. phaffii* BG10 strain), was used as the eukaryotic expression host [36]. To test the broader applicability of the CRISPR-MAD7 system, a previously made *K. phaffii* strain was taken from our collection. The strain used carries an integrative expression plasmid to produce a red fluorescence protein (*DsRed*) under the control of the constitutive *GAP* promoter (P*_GAP_*) and a Zeocin-resistance cassette with the Sh ble gene under the control of the constitutive *ILV5* promoter (P*_ILV5_*) [37].

### 2.3. Target Genes

The initial evaluation of the functionality of the CRISPR-MAD7 system in *K. phaffii* was made by targeting genes whose disruption can be easily detected using the phenotypic analysis of transformants (Table 2).

Additionally, 259 genes (Supplementary File S2, Table S1) encoding kinase and kinase-like proteins were chosen and targeted using CRISPR-MAD7 and CRISPR-Cas9 for comparison of the systems for a more significant number of target genes. The selection of kinase genes was based on the *K. phaffii* CBS7435 annotation by Sturmberger et al. [36].

### 2.4. gRNAs Design

The gRNAs were designed using the CCTop online tool [38]. The gRNA sequences were chosen to bind within the first quarter, downstream of the start codon, to the target genes and were selected randomly. The CRISPR-MAD7 gRNAs were 21 bp, and the selected PAM-site consensus sequence was 5′-YTTN-3′. For CRISPR-Cas9 gRNAs, the length of the gRNA was 23 bp, and the PAM site was 5′-NGG-3′. MAD7 gRNAs targeting *DsRed* and Sh ble were designed based on the sequences provided in Supplementary File S1 (S1 and S2). The 259 gRNAs for targeting the kinases are listed in Supplementary File S2, Table S2.

### 2.5. Assembly of the CRISPR-Cas9 and CRISPR-MAD7 Plasmids

**Figure 1:**
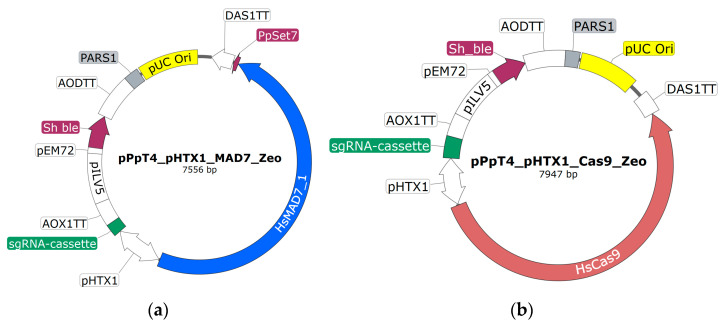
Schematic representation of the CRISPR-MAD7 (**a**) and CRISPR-Cas9 (**b**) *E. coli*/*K. phaffii* shuttle vectors. Vector (**a**) encodes the gene for the expression of MAD7 (blue), whereas (**b**) encodes the genes for the expression of Cas9 (red). In both cases, the transcription is initiated by one side of the bidirectional, constitutive RNA polymerase II promotor *HTX1* (P*_HTX1_*) and terminated by the *DAS1* terminator (*DAS1*TT). Transcription of the sgRNA cassette (green) is initiated by the second side of the P*_HTX1_* and terminated by the *AOX1* terminator (*AOX1*TT). For selection purposes in *E. coli* and *K. phaffii*, a Zeocin resistance gene (Sh ble, purple) under the control of P*_ILV5_* and P*_EM72_* is used. For transient expression of the CRISPR elements in *K. phaffii*, the plasmids harbor an autonomously replicating sequence (PARS1, grey). For plasmid propagation during cloning procedures in *E. coli*, an origin of replication (pUC Ori, yellow) is encoded.

The CRISPR-Cas9 plasmid made by Weninger et al. [15] served as base for the CRISPR-MAD7 (Figure 1a) and CRISPR-Cas9 (Figure 1b) vectors used in this study. The base plasmid is an *E. coli*/*K. phaffii* shuttle vector and comprises the *Cas9* gene with a C-terminal NLS and the sgRNA cassette, both under the control of the bidirectional polymerase II promoter *HTX1* (P*_HTX1_*) [39,40,41]. For transformant selection in both *E. coli* and *K. phaffii*, a Zeocine (Sh ble) resistance cassette driven by two promoters (P*_EM72_* and P*_ILV5_*) is used. For plasmid propagation in *E. coli*, a bacterial origin of replication (pUC) is used. To facilitate transient expression of the nuclease and the sgRNAs, the *Pichia* autonomously replicating sequence 1 (PARS1) is contained on the plasmid [15,42]. To facilitate faster cloning of gRNAs, two minor modifications were introduced into the CRISPR-Cas9 plasmid of Weninger et al. [15]: (i) *Bsmb*I and *Spe*I restriction sites in sgRNA expression cassette were added; (ii) 6 adenines at 5′ and 3′ ends of hammerhead ribozyme sequence were introduced (Supplementary File S1; S3). The sequences of the two vectors can be found in Supplementary File S1 (S4). SnapGene version 4.2.11 has been used for in silico work.

In order to optimize the CRISPR-MAD7 tool, different codon optimizations of the *MAD7* gene were tested (see Section 3.1). The *E. coli* codon-optimized *MAD7* sequence information had been published previously by Inscripta [43]. The codon optimizations of *MAD7* according to the average *Homo sapiens* and *K. phaffii* codon usage tables were performed using Gene Designer 1.1.4.1 (DNA 2.0 Inc./now ATUM, Newark, USA). For both optimization procedures, the codon usage tables included in the tool were used. However, in the case of the *H. sapiens* optimized *MAD7* genes, the optimization was completed four times to obtain four different distinct sequences (*H. sapiens* 1–4). The *MAD7* sequences used in this study are provided in Supplementary File S1 (S5).

To assemble the CRISPR-MAD7 vector, a CRISPR-Cas9 base plasmid (Figure 1b) lacking an sgRNA was used. Instead of the sgRNA, this plasmid had a *Not*I restriction site in between the P*_HTX1_* and the *AOX1*TT (Figure 1b). To replace *Cas9* with *MAD7*, the CRISPR-Cas9 base plasmid was PCR amplified using Primer 1 and 2 (Supplementary File S1, S6). The synthetic *MAD7* genes including the C-terminal NLS were designed to have 40 bp homologous overhangs to P*_HTX1_* and *DAS1*TT at the 5′- and 3′- end, respectively. Since *MAD7* is rather large (approx. 3.8 kb), the gene had to be ordered in three pieces which had a 40 bp overhang to each other. The different NLS sequences were included in the distal *MAD7* fragment. Thus, for each of the tested NLS an individual distal *MAD7* fragment was ordered. To assemble the CRISPR-MAD7 plasmid, Gibson cloning was used [20]. For plasmid assembly, the PCR linearized CRISPR-Cas9 plasmid was incubated with three appropriate *MAD7* fragments in the presence of the Gibson assembly mix at 50 °C for 45 min. Subsequently, 2.5 µL of the assembly mixtures were used for the transformation of competent *E. coli* XL1-blue cells. For transformant selection, the cells were plated on an LB-agar (Carl Roth, Karlsruhe, Germany) containing 25 µg/mL of Zeocin. The sequence of the CRISPR-MAD7 plasmids was confirmed using Sanger sequencing (Microsynth AG, Balgach, Switzerland).

To insert the three different sgRNA cassettes downstream of the P*_HTX1_*, the assembled CRISPR-MAD7 plasmids were linearized with *Not*I (Figure 1a). The sgRNA cassettes were ordered as synthetic DNA fragments and contained a 30 bp homology region for the 5′ promoter and the 3′ terminator elements of the plasmid. The plasmid assembly was completed using Gibson cloning as described above. Three distinct sgRNA cassettes with different gRNA scaffold sequences were designed and tested. The gRNA scaffold sequences published by Inscripta were either found in the FAQ section of the Inscripta website, or in the sections about yeast and *E. coli*, respectively. To facilitate efficient cloning of the gRNA fragments (see Section 2.6), a unique *Nhe*I restriction site is located downstream of the gRNA scaffold sequences. The sequences of the tested sgRNA constructs are given in Supplementary File S1 (S3). The plasmids were sequence verified using Sanger sequencing (Microsynth AG, Balgach, Switzerland).

In two cases in the below-described experimental section, the *K. phaffii* strain that should be modified had already a functional zeocin resistance cassette integrated into the genome. To be still able to select for positive transformed *K. phaffii* cells, a different selection marker gene than the Sh ble gene was needed on the CRISPR-MAD7 plasmids. Thus, CRISPR-MAD7 were completed plasmids with a Hygromycin B resistance gene (*HPH*) marker which can also be used for selection in *E. coli* and *K. phaffii*. To exchange the resistance gene, the CRISPR-MAD7 plasmid was digested with *Mfe*I and *Xba*I restriction enzymes. Subsequently, a synthetic *HPH* with 30 bp overhang to the upstream P*_EM72_* and downstream *AOD*TT (Figure 1b) was inserted using Gibson assembly cloning as stated above. The sequence of the plasmid was verified using Sanger sequencing (Microsynth AG, Balgach, Switzerland)

### 2.6. gRNA Gene Cloning

Two methods were used to insert the gRNA sequences into the sgRNA cassette on the CRISPR vectors, in vivo and in vitro recombination cloning. *E. coli* transformants were selected on LB-media containing either 50 µg/mL of Zeocin or 100 µg/mL of Hygromycin B depending on the plasmids used. The sequence of the plasmids was verified using Sanger sequencing (Microsynth AG, Balgach, Switzerland). Moreover, 44 CRISPR-MAD7 plasmids with specific gRNA coding sequences were ordered from Twist Bioscience (San Francisco, CA, USA).

#### 2.6.1. gRNA In Vitro Cloning

For the in vitro cloning, 60 bp ssDNA oligonucleotides were ordered (Supplementary File S1, S6 and Supplementary File S2, Table S3). The oligonucleotides contained either the target specific 21 bp or 23 bp gRNA for the CRISPR-MAD7 and the CRISPR-Cas9 system, respectively. The gRNAs were flanked by sequences identical to the gRNA scaffold sequence and the HDV ribozyme at the 5′- and 3′- end, respectively, for the MAD7-plasmids. The recommended oligonucleotide sequence for the CRISPR-MAD7 plasmid is GTCGGAATTTCTACTCTTGTAGAT(N)_21_GGCCGGCATGGTCCCAGC, and for the CRISPR-Cas9 it is 5′-AGTAAGCTCGTCAAAAAA(N)_23_GTTTTAGAGCTAGAAATAG-3′. To make CRISPR plasmids fully functional (N)_21_/(N)_23,_ which are gRNA sequences, had to be inserted. For that purpose, the CRISPR-MAD7 plasmid was linearized with *Nhe*I, and the CRISPR-Cas9 plasmid was linearized using *Bsm*BI and *Spe*I for Gibson cloning with a 50 nM final concentration of the respective oligonucleotides. Using this strategy, the CRISPR-MAD7 plasmids for targeting the initial nine genes and the CRISPR-Cas9 plasmids for targeting the 259 genes encoding for kinase were assembled.

#### 2.6.2. gRNA In Vivo Cloning

For in vivo cloning, the CRISPR-MAD7 vector was linearized with *Nhe*I. Subsequently, 1 ng of the linearized plasmid was used as the template for a PCR reaction using primers that would introduce a 21 bp homologic region that is used by *E. coli* for circularization during in vivo recombination. The reverse primer (5′-ATCTACAAGAGTAGAAATTCCGACGAGCTTACTCGTTTCGTCCTCACGGACTCATCAG-3′) was identical for all PCR reactions. In contrast, the forward primers were unique for each construct, as they contained the 21 bp gRNA which is specific for the target sequence (Supplementary File S2, Table S3). The PCR cycling parameters used for the amplification were 95 °C/5 min–(95 °C/20 s–55 °C/20 s–72 °C/8:30 min) × 25 cycles–72 °C/10 min. In the case of successful plasmid amplification, fragments of ~7600 bp were observed by agarose gel electrophoresis. Finally, 5 µL of the PCR reaction with a correctly amplified, linear plasmid was used to transform competent *E. coli* XL1-blue cells, which would result in the in vivo re-circularization of the plasmids. Using this technique, the CRISPR-MAD7 plasmids for targeting the 212 genes encoding for kinase were assembled.

### 2.7. Donor DNA Design

In total, 16 genes were selected as targets for HDR. For that reason, one double-stranded donor DNA fragment for each CRISPR-MAD7 and CRISPR-Cas9 plasmid was designed. The synthetic DNA fragments consisted of the 40 bp long identical insertion region plus, if necessary, two nucleotides to mutate PAM flanked by 130/130 or 130/128 bp regions homologous to the target gene. All DNA fragments, whose sequences are presented in the Supplementary File S2 (Table S4), were ordered from Twist Bioscience (San Francisco, CA, USA).

### 2.8. Transformation, Cultivation, and Screening

Transformation of electrocompetent *K. phaffii* cells was performed according to Lin-Cereghino et al. [44] using 100 ng (~20 fmol) of circular plasmids and 60 ng (~310 fmol) of corresponding donor DNA fragments. Transformants were plated on YPD (1% *w*/*v* yeast extract, 2% *w*/*v* peptone, and 2% *w*/*v* glucose), or YP supplemented with 2% *v*/*v* ethanol when targeting *TPI*, plates (1.5% agar). For selection of positive transformants, the plates either contained 100 µg/mL Zeocin or 300 µg/mL Hygromycin B, depending on the CRISPR plasmid employed, were used. Transformation plates were incubated at 28 °C for three days. When targeting *TPI1*, *PMT2*, and *OCH1*, the incubation time was increased to 6+ days due to slow growth rates. Transformants were cultivated in 96-deep-well plates (Bel-Art Products, Pequannock Township, NJ, USA) with 250 µL YPD for 24 h (*GUT1*) or 60 h (for *OCH1* and *PMT2*) or 250 µL YP-ethanol for 60 h (*TPI1*) at 320 rpm and 28 °C. When targeting kinases, four to eight transformants per target were randomly picked and cultivated in deep well plates with 250 µL YPD containing 100 µg/mL Zeocin for two days at 28 °C and 320 rpm.

To screen for the disruption of *GUT1* and *TPI1*, cell material was either transferred from the DWP onto buffered minimal glycerol (1.34% YNB, 1% glycerol, 4 × 10^−5^% biotin, 200 mM potassium phosphate buffer, pH 6.0) and YPD plates (*GUT1*) or YP-ethanol plates (*TPI*) using a metallic stamp. For detecting Sh ble disruption, cultivations were stamped onto YPD and YPD selection plates containing 100 µg/mL Zeocin. The agar plates were incubated at 28 °C for two days. To screen for *DsRed* disruption, the fluorescence of a 1:20 dilution of the deep well cultivation with ddH_2_O was measured (exciting wavelength 554 nm and detection wavelength 581 nm). *PMT2* and *OCH1* cultivations were stamped on YPD plates and incubated for multiple days at 28 °C.

**Figure 2:**
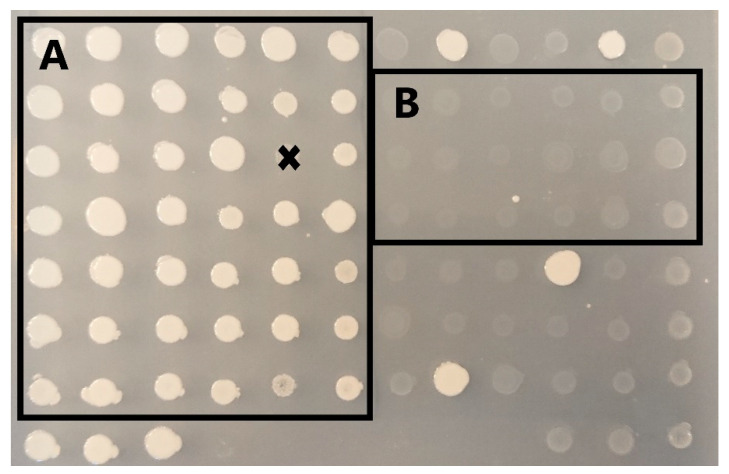
Screening plate for *GUT1* deficient *K. phaffii* clones. After transformation with the CRISPR-MAD7 vectors, *K. phaffi* transformants were cultivated in 96-deep-well plates containing 250 µL YPD media for 48 h at 28 °C and 320 pm. Cell material from the cultivations was transferred onto screening plates with buffered minimal media plates containing 1.34% YNB, 4 × 10^−5^% biotin, 200 mM potassium phosphate buffer (pH 6.0), and 1% glycerol as carbon source using a metallic stamp. After 48 h at 28 °C, the number of mutants with a reduced growth phenotype on the glycerol media was evaluated: Clones in box A show wildtype-like growth (with the exception of one spot that was marked with an x to be excluded from this visualization); box B represents mutants with reduced growth, which are regarded as *GUT1* deficient in this experiment.

To calculate the editing efficiencies based on phenotypic characteristics when the *GUT1* and Sh ble were targeted, the colonies showing normal and reduced/non growth when they were cultivated on BMG and YPD + Zeocin screening plates, respectively, were counted (Figure 2). The targeting efficiency for *DsRed* was determined as the ratio of clones with wildtype-like fluorescence levels to clones with fluorescence levels like the control strain carrying the *DsRed* expression cassette.

To confirm editing of the target genes on the DNA level, each target gene was amplified using colony PCR using the Phire Plant Direct PCR Master Mix (Thermo Fisher Scientific (Vienna, Austria)). Subsequently, the PCR product, if the correct amplicon was observed using agarose-gel electrophoreses, was sent for Sanger sequencing (Microsynth AG, Balgach, Switzerland). Sequence results were aligned to corresponding genes to detect the presence or the absence of indels. For PCR preparation, 5 µL of the cell culture of each single clone cultivated into a DWP was mixed with 45 µL of the Dilution buffer (supplemented with Phire Plant PCR Master mix). For the colony PCR, 5 µL of diluted samples were used as a template, and the PCR conditions were set according to the manufactures’ protocol. In case the *GUT1*, Sh ble, *DsRed*, *TPI1*, *PMT2* and *OCH1* were targeted, all colonies showing phenotypical characteristics were amplified and sequenced. To confirm the editing of the kinases, 4–8 colonies per target were screened. The primers used for amplification of the *GUT1*, Sh ble, *DsRed*, *TPI1*, *PMT2*, and *OCH1* can be found in Supplementary File S1 (S6), and the ones used for amplification of the kinase genes are provided in Supplementary File S2 (Table S5).

## 3. Results

### 3.1. Setting Up CRISPR-MAD7

Based on the previous experience of establishing a functional CRISPR-Cas9 system in *K. phaffii* [15], it was known that codon optimization of the nuclease, the employed NLS sequence, and the gRNA scaffold sequence are the key to a good working genome-editing tool. Thus, 30 combinations of different *MAD7* codon optimizations, NLS, and gRNA scaffold sequences were tested to find the combination resulting in the best genome-editing efficiency (Table 3). In total, six different codon-optimized synthetical *MAD7* were ordered. We tested (i) the *E. coli* optimized sequence of Inscripta, (ii) a *K. phaffii* optimized gene, and (iii) four genes using different codon optimizations for *Homo sapiens*. Several NLS were tested: (i) the *S. cerevisiae* Setp7 (syn. Rkm4), a ribosomal lysine methyltransferase, homolog in *K. phaffii* (*Pp*Set7); (ii) the Simian Virus 40 large T antigen (SV40, also used by Weninger et al. [15]); and (iii) the *Xenopus laevis* nucleoplasmin sequence (*Xl*Nuc) [45]. The NLSs were chosen as they should facilitate different translocation levels of the nuclease to the nucleus, as they did for eGFP in a previous study [45]. Similarly, three distinct gRNA scaffold sequences that facilitate the binding of the nuclease to the gRNA were tested (Supplementary File S1, S3). The gRNA scaffold sequences were obtained from the website of Incripta and were either found in the FAQ section with apparently no specific assignment to a host organism [43] or were specifically designated for their use in yeast [46] and *E. coli* [47], respectively.

To evaluate the performance of the different constructs, we targeted the *GUT1* locus using the same gRNA based on a 5′-TTTA-3′ PAM site (Supplementary File S1, S7). Since disruption of the *GUT1* results in phenotypes with reduced growth when cultivated on a medium containing glycerol as the sole carbon source (Figure 2), we could calculate the ratio of slow-growing phenotypes obtained from the randomly selected transformants to which we refer to in the text as disruption efficiency.

The initial studies were performed using the *E. coli* and *K. phaffii* optimized *MAD7* in combination with the three distinct NLS and gRNA scaffold sequences each (Table 3, Construct 1–18). Evaluating the efficiency of the different CRISPR-MAD7 constructs showed that the *K. phaffii* codon-optimized *MAD7* gene did only work as a functional nuclease when combined with the SV40 and the *E. coli* gRNA scaffold sequence (Table 3, construct 14). All other constructs using the *K. phaffii* codon-optimized *MAD7* did not result in slow-growing phenotypes, and hence no genome-editing events occurred (Table 3). Despite the non-functionality of most tested *K. phaffii* optimized *MAD7* constructs, one functional construct still resulted in a gene disruption efficiency of 45%. In the case of the *E. coli* optimized *MAD7*, the highest gene disruption efficiency (71%) was reached in combination with the *Pp*Set7 and the *E. coli* gRNA scaffold sequence (Table 3, Construct 8).

To see if the efficiency of the tool could be increased, we tested in the next step the *H. sapiens* codon-optimized MAD7 nucleases in combination with different NLS. This was completed because it was shown previously that the *H. sapiens* optimized Cas9 worked best in *K. phaffii* [15]. To reduce the number of variables, and since based on the results obtained in this study the *E. coli* gRNA scaffold sequence worked best (Table 3), we decided to only use this gRNA scaffold sequence for testing the *H. sapiens* optimized *MAD7* genes. Assessing the functionality of the 12 constructs (Table 1, Construct 19–30) suggested that *H. sapiens* optimized *MAD7* genes work better than the *E. coli* and *K. phaffii* codon optimization of *MAD7*, as target disruption efficiencies of up to 90% were reached (Table 3, Construct 21). However, like before, large differences between the codon usages and the NLS sequences employed were observed. It appears that *H. sapiens* codon usage 1 works best, as all constructs resulted in *GUT1* disruption. Still, we observed a large variation in the disruption efficiency depending on the NLS we used (3% *Xl*Nuc vs. 90% *Pp*SET). The *MAD7* genes optimized according to *H. sapiens* codon usage 2, 3, and 4 did not result in target disruption in combination with the SV40 but seemed to work in combination with the *Pp*SET7 (Table 3). Nonetheless, they do not reach the 90% *GUT1* disruption efficiency and were thus not considered for further studies.

Summing up, the most efficient CRISPR-MAD7 system for application in *K. phaffii* was obtained by combining the *MAD7* gene optimized according to *H. sapiens* codon usage 1 (Table 3) with the *Pp*SET7 NLS and the *E. coli* gRNA scaffold sequence (Table 3, Construct 21). This construct resulted in a *GUT1* disruption efficiency of ~90% which was close to the previously reported CRISPR-Cas9 system for *K. phaffii* [15]. Moreover, our data allow for speculations that the key to the efficiency of construct 21 is low transcript levels and low levels of nucleases in the nucleus. This assumption is based on the finding that constructs with *MAD7* optimized according to the distantly related codon usages, which should result due to the codon bias in lower transcript levels [48], work better than the *K. phaffii* codon usage. This is in line with the observed fewer *K. phaffii* transformants when the plasmid carrying the *K. phaffii* optimized *MAD7* was used for transformation (Table 3, Construct 10–18), suggesting there is stress and potential lethality caused by off-targeting due to increasing the amounts of nuclease [49,50].

### 3.2. 5′-TTTN-3′ PAM Sites Are Favored in K. phaffii

One of the critical elements when designing gRNA is choosing the correct PAM site. In the case of MAD7, the consensus sequence of the PAM site is 5′-YTTN-3′ in which “Y” can be either a “T” or “C”, and “N” can be one of the four standard nucleotides. To see which PAM site works best for genome engineering using MAD7 in *K. phaffii*, gRNAs based on all eight possible PAM sequences were tested (Table 4). To investigate the potential influence of the gRNA binding site on disruption efficiency, two gRNAs were tested per PAM site sequence (Table 4). Identical to the previous experiment, we targeted the *GUT1*, as gene disruptions can be easily monitored (Figure 2). Although Construct 21 works best for *GUT1* disruption, we decided to use construct 8 (Table 3) for assessing the influence of the PAM site, as the disruption rate of 71% allows for the detection of higher and lower target disruption efficiencies.

Our results indicate an influence of the PAM site on *GUT1* disruption efficiency. It appears that the 5′-“Y” in the PAM sequence needs to be occupied by a “T” in *K. phaffii* as the constructs based on “C” did not work for gene disruption (Table 4, #9–16). When looking at the 3′-“N”, our data indicate differences between the PAM sites and might even show that PAM sites can improve the overall efficiency of the CRISPR-MAD7 tool, but due to the experimental set-up (no biological replicates), we cannot say this for certain (Table 4). Still, we decided to stick to 5′-TTTN-3′ PAMs for subsequent studies, as they seem to work best for CRISPR-MAD7 in *K. phaffii*.

Besides the influence of the PAM site sequence on the gene-editing efficiency, we can confirm previous observations [51,52] that the gRNAs design is critical for gene disruption, as in two cases only one of the two tested gRNAs per PAM site sequence was functional (Table 4).

Of note, although the gRNA for targeting the *GUT1* locus in the initial study to set up the CRISPR-MAD7 system (Table 3) is based on a 5′-TTTA-3′ PAM, the gRNA was different from #1 and #2 in Table 4. Thus, the difference in target disruption efficiency (79% vs. 21%) is not caused by experimental flaws but supports our point that the gRNA is of major relevance for the efficiency of the CRISPR-MAD7 system. Additionally, further factors like the intra-genetic gRNA target locus, the accessibility of the target sequence, and the position-specific sequence features of the target nucleic acid may also impact the efficiency, as outlined previously [52,53]. Therefore, it is suggested that it would be helpful to design and test multiple gRNAs per target gene and to screen a large and diverse set of transformants to increase the chances of successful gene editing.

### 3.3. Using CRISPR-MAD7 to Edit Other Targets Than the GUT1 Gene

The *GUT1* gene is a convenient target for testing and establishing the CRISPR-MAD7 system in *K. phaffii*, since it is easy to screen for gene disruption events and the locus seems to be easily accessible for genome engineering procedures. Nonetheless, to verify the system’s robustness and broader applicability, additional integrated genes in the *K. phaffii* genome were targeted and aimed to be disrupted by the established CRISPR-MAD7 tool (Table 3, Construct 21).

As a next gene, the triosephosphate isomerase (*TPI1*) gene was targeted, a central enzyme in glycolysis, that is responsible for the interconversion of dihydroxyacetone phosphate (DHAP) to D-glyceraldehyde 3-phosphate (GAP). Due to its central role in carbon source metabolism, it can on the one hand be considered as a challenging disruption target, because *TPI1* deficient *S. cerevisiae* strains show poor growth on most carbon sources, which means there would be a selection for functional Tpi1p on transformation plates [54,55], and, on the other hand, it could be used as an interesting knockout target due to the potential use of *TPI1* as selection marker in a *TPI1* deficient host strain [56,57]. In addition, several other research teams reported before that such a knock out was not possible before in *K. phaffii* (personal communication). Successful *TPI1* inactivation would result in slow growth on glucose and no growth on glycerol as a carbon source and has thus considerable impact on the cell metabolism [54,57]. To edit the *TPI1* using the CRISPR-MAD7 system, we designed and tested four distinct gRNAs (Supplementary File S1, S7) to increase the chances of a successful CRISPR-MAD7 construct. gRNAs 1-3 led to potential *K. phaffii* ∆*tpi1* strains. The potential knockout mutants were identified, as they showed, in line with the premise that *TPI1* is essential for glycerol utilization [57], no growth on glycerol as the sole carbon source. To investigate if indeed the inactivation of *TPI1* is responsible for the inability of these strains to utilize glycerol, the *TPI1* gene was PCR amplified and sequenced. The genotyping showed, as expected, frame-shifting deletions, confirming successful *TPI1* inactivation with CRISPR-MAD7.

The second alternative *K. phaffii* target was the *OCH1* gene*. OCH1* encodes for α-1,6-mannosyltransferase, which catalyzes the first step of the hypermannosylation of proteins in the Golgi apparatus in *K. phaffii* (and other yeasts) and is, therefore, critical for the glycosylation levels of secretory proteins [58,59]. Thus, the inactivation of *OCH1* results in the reduction of mannosylation levels, easing downstream processing and increasing the biopharmaceutical properties of secreted proteins ([60,61]) which makes it a highly appreciated target. However, *OCH1* is known from previous genome-engineering efforts as a target with low disruption efficiencies [58], making it a challenging locus for testing the established CRISPR-MAD7 system. As for the other targets, we were able to inactivate *OCH1* using the MAD7-based CRISPR tool. Still, in the case of *OCH1*, we could only observe the characteristic *OCH1* phenotype featuring reduced cell wall integrity and growth as outlined by Krainer et al. [58], when gRNA 1 (Supplementary File S1, S7) was used to direct the MAD7 nuclease to the *OCH1* locus highlighting again the importance of the gRNA. The disruption of the *OCH1* reading frame was subsequently confirmed by sequencing the PCR-amplified *OCH1* coding genomic fragment.

Another challenging target for testing the CRISPR-MAD7 system was found in *PMT2*. In fungi, protein-O-mannosyltransferases (PMTs) are responsible for protein-O-mannosylation in the ER, and *PMT2* is the highest expressed representative [62]. While a knockout of the *PMT2* seems to be lethal for *Schizosaccharomyces pombe* and *Candida albicans*, it was reported in *K. phaffii* to lead to the secretion of proteins with a reduced O-glycan site occupancy and O-glycan chain length [62]. This suggested that the *PMT2* is a difficult but very valuable knockout target, as it brings similar benefits for the secretion of proteins like a *OCH1* knockout [62,63,64]. Like for *OCH1*, only one of the six gRNA (gRNA 6, Supplementary File S1, S7) resulted in editing of the *PMT2* using the CRISPR-MAD7 tool. The multiple potential ∆*pmt2* mutants were identified using their slow-growing phenotype as previously described [62]. However, when we evaluated the sequencing results to confirm the *PMT2* knockout, we discovered that the slow-growing phenotypes had short frame-shifting indels as well as very large frame-shifting (>1000 bp) and in-frame deletions (363 bp) meaning such deletions were sufficient to alter the Pmt2p activity enough to result in phenotypical characteristics. This supports the observations that *PMT2* is of high importance for *K. phaffii* or might be connected to the nuclease itself as discussed in the following section.

At this point, it has to be mentioned that, although we could knockout the *TPI*, *OCH1* and *PMT2* using the CRISPR-MAD7 system, the gene disruption efficiency was low, most likely caused by the importance of genes for cellular integrity. Indeed, without pre-screening for the anticipated phenotypical characteristics, such as slow growth, and testing a variety of gRNAs it would be difficult to identify respective knockout strains among other colonies of non-modified cells. Thus, we thought it was misleading to calculate the target disruption efficiencies of the MAD7 system based on those challenging targets.

Thus, we decided to use a previously made *K. phaffii* strain, expressing the red fluorescence protein (*DsRed*) as the reporter protein, and carrying a Zeocin (Sh ble) resistance cassette for selection, as an alternative knockout target [37] to the *GUT1* gene. Like in previous experiments, two gRNAs per target were designed and tested (Supplementary File S1, S7), and the knockout candidates were picked randomly without pre-screening. To detect successful disruption of *DsRed* and Sh ble, the obtained clones were screened for their fluorescence levels and their ability to grow on media containing Zeocin, respectively. In accordance with our previous observation, we saw differences between the gRNAs employed for directing the nuclease to the target. In the experiments where *DsRed* was the knockout target, 32 out of 42 (76%) screened clones targeted by gRNA 1 and 30 out of 42 (71%), targeted by gRNA 2, showed wildtype-like fluorescence levels suggesting that the *DsRed* gene was disrupted (Table 5). Similar numbers were obtained when Sh ble was chosen as the knockout target, as 29 out of 42 (69%) clones targeted with gRNA 1 and 32 out of 42 (76%) screened clones targeted with gRNA 2 lost their ability to grow on Zeocin supplemented media (Table 5). This confirms that the CRISPR-MAD7 system is applicable for efficient ORF disruptions of native and non-native genes in *K. phaffii*.

### 3.4. Systematic Evaluation of the Genome-Wide-Editing Efficiency of the CRISPR-Cas9 and the CRISPR-MAD7 Systems in K. phaffii

Having access to a CRISPR-MAD7 system (Table 3, Construct 21), which showed the high gene-editing performance in *K. phaffii* for targeting various genes and previously Weninger et al. developed, by now the well-established, CRISPR-Cas9 tool [15], a comparison of the two systems as tools for high-throughput knock-out generation was initiated. For this purpose, 259 genes encoding kinases were selected, as it was previously shown that some kinases can be successfully disrupted in *K. phaffii* using classical genome engineering techniques [65], although a significant number of those regulatory proteins can be assumed to be essential for *K. phaffii*. Furthermore, the high number of target genes decreased the bias that comes from gRNAs’ design or the position of individual genes at chromosomes. Therefore, it enabled determination of a genome-wide-editing efficiency, which can be applied to most of the genes of both systems.

Since Cas9 and MAD7 require different PAM sites, two distinct sets of target specific gRNAs (Supplementary File S2, Table S2) had to be designed for each CRISPR system. 5′-NGG-3′ and 5′-TTTN-3′ were used as consensus sequences of PAM sites Cas9 and MAD7, respectively.

Although in the experiments targeting *GUT1*, *DsRed*, and Sh ble genes the disruption efficiency was defined as the ratio of the clones with edited genes to the total number of screened clones in which the same genes were targeted, the different approaches for defining the gene-editing efficiency were used in the comparison study. As we searched for a parameter that characterizes the average efficacy of two CRISPR systems, the genome-wide-editing efficiency was calculated as the ratio of the number of edited genes to the total number of targeted genes.

In total, 181 of the 259 (70%) kinase genes were successfully mutated. In total, 169 of 259 (~65%) targeted kinases were edited when the CRISPR-Cas9 system was used, and 59 of 259 (~23%) genes were edited by employing the CRISPR-MAD7 system (Table 6, Supplementary File S2, Table S6, Supplementary File S3). A total of 47 of the kinases were mutated using both tools. Consequently, 122 genes could only be mutated using Cas9, and 12 only using MAD7. Thus, the data indicated that kinase open reading frames can be successfully engineered using both CRISPR methods, but the genome-wide-editing efficiency of the CRISPR-Cas9 was approximately three times higher than the genome-wide-editing efficiency of the employed CRISPR-MAD7 system.

### 3.5. Editing Using the CRISPR-MAD7 System Resulted in a Higher Percentage of In-Frame Mutations

Intrigued by the low editing efficiency of the CRISPR-MAD7 system, we further scrutinized the sequencing results. It appears that the probability to obtain in-frame mutations depends not only on the targeted gene [66], but also on the nuclease used for editing itself. According to our data, 36% (21 of 59) of the kinases edited using the CRISPR-MAD7 system had in-frame mutations. In contrast, the editing events caused by the CRISPR-Cas9 only resulted in 16% (27 of 169) of in-frame indels (Table 6) after repairing double-strand breaks using non-homologous end joining. In the genes edited using both systems, CRISPR-Cas9 generated frame mutations in only ~6% (3 of 47) of the editing events, whereas CRISPR-MAD7 induced an in-frame indel in 36% (17 of 47) of the editing events. Hence, it appears that the gene editing of the *K. phaffii* genome using the CRISPR-MAD7 is more likely to lead to in-frame mutations than editing using the CRISPR-Cas9.

This observation fits well with the fact that Cas9 and MAD7 belong to different nuclease families with different cleavage patterns [17]. MAD7, belonging to the Cas12a family, induces PAM-distal cleavage with a 5′ overhang of variable length [17]. In addition, it was suggested that Cas12a could tolerate small indels created using NHEJ, leading to multiple rounds of DSB formation, as such enhancing the chance of HR occurrence [17]. Unlike Cas12a, Cas9 endonuclease creates blunt ends or a short overhang and after NHEJ repair Cas9 does not cleave the altered target sequence [17].

### 3.6. Identification of Favorable PAM Sequences for the CRISPR-MAD7and the CRISPR-Cas9 Systems in K. phaffii

The preliminary testing of the CRISPR-MAD7 system indicated that the PAM site used for gRNA design might affect the target disruption efficiency of the MAD7 endonuclease but did not show a clear trend (Table 3). In addition, it was recently reported that the PAM site can influence the cleavage efficacy of the CRISPR-Cas9 system [67]. Thus, we performed some simple calculations to test the influence of the PAM sites in a larger setup and to identify/confirm preferable PAM sites for both systems.

For the calculations, the gRNAs used in the CRISPR-MAD7 system were classified into four groups according to the PAM sequences. In our study, the least-selected PAM site sequences from the CCTOP tool were 5′-TTTA-3′ (14%) and 5′-TTTT-3′ (21%), whereas the other two PAM sites (5′-TTTG-3′ and 5′-TTTC-3′) were chosen from the tool with similar probability (~34% and ~31%, respectively). Subsequently, the absolute and relative numbers of gRNAs and editing events were calculated for each group. The gRNAs which were designed based on the 5′-TTTC-3′ and 5′-TTTA-3′ PAM-site sequences resulted in editing efficiency rates of 28% and 27%, respectively. The 5′-TTTT-3′ and 5′-TTTG-3′ PAM-site sequences had editing efficiencies of 19%, and were slightly less efficient but still very comparable (Table 7). Thus, we could confirm the trend observed in Table 4 suggesting that all four 5′-TTTN-3′ based PAM sites are suitable to obtain gene knockouts in *K. phaffii* using the CRISPR-MAD7 tool.

Similarly, as for the MAD7-based system, the gRNAs used in the CRISPR-Cas9 system were grouped according to the 5′-NGG-3′ PAM consensus sequences into four classes. In this case, the 5′-AGG-3′ (32%) and 5′-TGG-3′ (40%) based gRNAs occurred more frequently than the 5′-CGG-3′ (15%) and 5′-GGG-3′ (13%) based gRNAs. Consequently, the 5′-AGG-3′ and 5′-TGG-3′ based gRNAs were involved in the majority of the observed editing events (Table 8). However, the calculation of the average gene-editing efficiency for each gRNA revealed that using 5′-CGG-3′ and 5′-GGG-3′ based gRNAs result in ~76% of cases in an editing event. In contrast, 5′-AGG-3′ and 5′-TGG-3′ based gRNAs appear to be less efficient, as the gene-editing efficiencies observed were 61 and 62%, respectively (Table 8). Thus, the obtained data suggested that “C” and “G” were the preferred occupations for “N” in the 5′-NGG-3′ PAM consensus sequences in *K. phaffii* for the CRISPR-Cas9 system, but, like for the CRISPR-MAD7, the differences in editing efficiency observed between the used PAM sites is not large and will most likely not be the key for an gRNA to be functional or not.

### 3.7. Comparison of HDR-Mediated Gene Editing of CRISPR-MAD7 and CRISPR-Cas9

The obvious contradiction between the results obtained during the initial establishment and the genome-wide testing of the CRISPR-MAD7 platform needed further investigation. Although the differences in sample sizes may be a possible explanation for the contradiction, another obvious hypothesis was that the staggered ends resulting from the MAD7 cutting mechanism might be less prone to errors when microhomologies are present or repaired using a simple ligase process rather than a non-homologous end-joining process of blunt-ended fragments after double-strand breaks caused by the Cas9 nuclease. If true, this phenomenon could be masking the actual genome-wide-editing efficiency of the CRISPR-MAD7 system. Despite the fact that microhomology-mediated end joining (MMEJ) is considered an error-prone repair mechanism in the literature [68,69], investigation of the above-mentioned hypothesis seemed worthwhile.

In order to investigate this hypothesis, it was decided to try to detect DSB events, if they indeed take place, that might be triggered by CRISPR-MAD7 plasmids which did not provide indels in the genome-wide-editing study. However, this time, the occurrence of DSB was checked not by looking for indels caused by NHEJ repair but by seeking knock-in events provided by HDR. For that reason, in addition to BSYBG10, the BSYBG10 Δ*ku70* strain (bisy GmbH, Hofstaetten an der Raab, Austria) was used in this experiment. This strain is known for its significantly reduced ability to repair DSB using NHEJ and due to the fact it favors HDR with donor DNA [14].

First of all, seven CRISPR-MAD7 plasmids targeting different genes and, based on previous experiments, that were considered non-functional (not able to cause indels) were selected. In parallel, seven functional CRISPR-Cas9 plasmids for the same genes were picked as positive controls which proved that knock-in events are possible in the chosen target genes. Both sets of plasmids and the corresponding donor DNA fragments were used for co-transformation of the *K. phaffii* strains BSYBG10 and BSYBG10 Δ*ku70*. After that, between four and seven clones from each transformation were analyzed to detect the occurrence of NHEJ- or HDR-mediated editing events. Results obtained during this experiment are presented in Table 9 under the subtitle “Main group” and they indicate that the CRISPR-MAD7 plasmids whose usage did not lead to indels’ generation are also not able to edit genes through HDR (0/7). As for the CRISPR-Cas9 plasmids, they successfully provided HDR integration events in BSYBG10 Δ*ku70* (7/7), though relatively short homologous arms and a low donor template concentration were used [70]. Since the MAD7 system had never been used for HDR-mediated integration, an additional control group including functional CRISPR-MAD7 and CRISPR-Cas9 plasmids was necessary.

For the control group, nine genes and, corresponding with them, functional CRISPR-MAD7 and CRISPR-Cas9 plasmids were selected. As is the case with the main group, BSYBG10 and BSYBG10 Δ*ku70* were transformed with CRISPR plasmids, and the donor DNA fragments, and the same analysis was completed. The summary of the results are presented in Table 9 under the subtitle “Control group”. Although the functional CRISPR-MAD7 plasmids indeed generate indels in BSYBG10 (8/9), their efficiency in HDR-mediated integration in the Δ*ku70* strain is noticeably lower (4/9). This phenomenon points out that the CRISPR-MAD7 system might be sensitive to some experimental conditions such as the concentration of donor DNA [70]. In contrast, the CRISPR-Cas9 demonstrated high performance under the current experimental conditions, and small fluctuations in HDR-mediated editing in Δ*ku70* (7/9) can be caused by the small number of screened clones.

To sum up, the experiment empirical evidence seriously undermined the initial hypothesis linking the staggering ends with a high chance of errorless DSB repair, but still enabling relatively efficient repair and gene editing using HDR using donor DNA. Despite the fact that the sample size and the number of analyzed transformants was quite limited, these data confirm the objectivity of the previously established genome-wide-editing efficiency of the CRISPR-MAD7 platform. In addition, the advantage of *ku*70 deletion strains for homologous recombination was confirmed, as well as their disadvantage for mutagenesis using error-prone NHEJ repair. Additional information including the number of edited clones for each individual targeted gene is available in Table S7 (Supplementary File S2).

## 4. Discussion

In this paper, the CRISPR-MAD7 system of Inscripta was adapted and systematically tested for the first time for use for *K. phaffii* genome engineering. Several codon optimizations for the *MAD7* gene in combination with a variety of NLS and gRNA scaffold sequences were tested. We discovered that the best tool is formed by combining a *H. sapiens* codon-optimized *MAD7* gene with the *Pp*SET7 NLS and the *E. coli* gRNA scaffold sequence of Inscripta. For establishing and assessing the applicability the MAD7 based tool, a variety of genes (Sh ble, *DsRed*, *PMT2*, *OCH1*, *GUT1*, *TPI1)* were targeted, and very favorable gene disruption efficiencies of ~90% could be obtained. This disruption efficiency has to be considered as a chance to detect a clone with an indel when a functional CRISPR-MAD7 plasmid was used. However, a more significant genome-wide knockout study in which 259 genes encoding for kinases were targeted with the CRISPR-MAD7 tool and the previous established CRISPR-Cas9 system indicated that the MAD7 based system is about three times less efficient then the Cas9 based system (~23% vs. ~65%).

Previous studies reported that the knockout efficiency of the CRISPR-Cas9 system in *K. phaffii* was over 90% [16,33]. Nevertheless, our genome-wide kinase knockout investigation indicated a lower editing efficiency of the CRISPR-Cas9 tool in *K. phaffii* as only around 65% of the attempted knockouts were successful. However, these differences might be explained by the dissimilarity between the CRISPR-Cas9 constructs used in the studies (e.g., different promotors were employed for the expression of the Cas9 gene and sgRNA, and different gRNA length) compared to our study but are most likely due to the fact that the number of essential genes in the selected group of central regulatory genes can be assumed to be high. Furthermore, it is also known that the efficiency of the CRISPR-Cas9 tool depends on factors like the gRNA sequence, GC content, and the accessibility associated with the chromatin state of the target sequence, and, for example, high nucleosome occupancy at a target site can prevent the access of CRISPR-Cas9 to the site [71,72,73]. Moreover, some genes encoding kinases might be vital for the cellular integrity of *K. phaffii*, making it also difficult or impossible to inactivate the functionality of such genes [65]. In fact, it is most likely a combination of all of the above-mentioned factors which makes it difficult to compare the gene-editing efficiency observed in this work and other studies. In further studies, it might be interesting to reproduce the knockout experiment but for another group of genes and/or to test the gene-editing efficiency of other/adapted CRISPR-Cas9 constructs for the kinases used in this study.

For the CRISPR-MAD7 system, the determined genome-wide-editing efficiency was around 23%. Despite the fact that there are no available data about the application of MAD7 in *K. phaffii*, in our opinion it is valid to compare our observations with the performance of the CRISPR-Cas12a system that was recently reported by Zhang et al. for genome engineering in *K. phaffii* [34]. In their study, the CRISPR-Cas12a editing efficiency for the four target genes using NHEJ repair varied between 90% and 10%, and the average efficiency was around 40% [34]. These numbers are in line with the noticeable variation in the editing efficiency of the CRISPR-MAD7 system observed in our study when the initial MAD7 testing on the limited number of targets demonstrated the high efficacy (~70%) of the tool, and extensive testing resulted in a low disruption efficiency (~23%). Although it is known that the staggered ends tended to be repaired through the error-prone MMEJ pathway [74], Cas12a-type nucleases may also generate DSBs whose repair can be error-free [75]. Based on that, we hypothesized that the ends after the cleavage using MAD7 might also favor accurate repairing, and due to that, some indels were not observed in the genome-wide screening. However, this hypothesis was not confirmed empirically. Nevertheless, it is not excluded that under other conditions the editing efficiency of the CRISPR-MAD7 system in *K. phaffii* can be improved. For example, it was reported that temperature affects the efficacy of Cas12a proteins, and at 37 °C they usually perform better than at 28 °C [76]. Another option is to find a new configuration of the promotor and terminator to increase the expression of *MAD7* because it was recently shown that the expression level of Cas12a positively correlates with the editing efficiency of the CRISPR-Cas12a system [77].

One more interesting aspect revealed by our research is that editing using the MAD7 system results in a remarkably higher number of in-frame mutations in comparison to the Cas9- based tool which is potentially connected with the pattern using which MAD7 cleaves the DNA. For gene editing employing HDR, with co-transformed donor DNA the difference between the two CRISPR systems became significantly smaller and larger target numbers would be necessary to draw a final conclusion. However, in cases of gene editing in strains which did not contain a *ku*70 deletion, the MAD7 system showed advantages which seems to be reasonably explained by the generation of long sticky ends in case there are double-strand breaks caused by the MAD7 nuclease. Such single-stranded DNA seemingly can replace dependence on an engineered strain background for efficient CRISPR based gene editing employing HDR. However, a larger number of targets needs to be analyzed to evaluate if there is a significant advantage of MAD7 for HDR in such wt backgrounds.

In conclusion, CRISPR-MAD7 is a suitable alternative to CRISPR-Cas9 for genome engineering in *K. phaffii*, as several selected targets could be edited. Based on the findings of this study, we recommend CRISPR-Cas9 as a preferable tool for gene disruptions due to double-strand breaks in combination with error-prone NHEJ repair in *K. phaffii*. And, CRISPR-MAD7 showed advantages for gene editing employing HDR with donor DNA fragments in the platform strain without *ku*70 deletion. In this study also, 181 *K. phaffii* strains with defined mutations in kinase genes were generated, which will be used for further investigations regarding their physiological functions and phenotypical consequences.

## Figures and Tables

**Table 2:** Target genes for setting up the CRISPR-MAD7 system.

Gene Name	Sequence Source
Red fluorescence protein (*DsRed*)	Vogl et al., 2018 [37]
Bleomycin resistance protein (Sh ble)	Vogl et al., 2018 [37]
Glycerol kinase 1 gene (*GUT1*)	NCBI (SOP83100.1, CAH2450543.1)
Triosephosphate isomerase (*TPI1*)	NCBI (SOP81882.1, CAH2449166.1)
α-1,6-mannosyltransferase (*OCH1*)	NCBI (SOP78939.1, CAH2445972.1)
Protein-O-mannosyltransferase 2 (*PMT2*)	NCBI (SOP81376.1, CAH2448629.1)

**Table 3:** Editing efficiencies of MAD7 and sgRNA constructs at the *GUT1* locus.

	CRISPR-MAD7 Constructs				Screening	
Construct	Codon Optimization	NLS	gRNA Scaffold	Total Number of Screened Clones	Number of Clones with Reduced Growth	Disruption Efficiency [%]
1	*E. coli*	*Xl*Nuc	Yeast	42	1	2
2	*E. coli*	*Xl*Nuc	*E. coli*	42	0	0
3	*E. coli*	*Xl*Nuc	Inscripta	42	14	33
4	*E. coli*	*SV*40	Yeast	42	0	0
5	*E. coli*	*SV*40	*E. coli*	42	0	0
6	*E. coli*	*SV*40	Inscripta	42	2	5
7	*E. coli*	*Pp*Set7	Yeast	42	22	52
8	*E. coli*	*Pp*Set7	*E. coli*	42	30	71
9	*E. coli*	*Pp*Set7	Inscripta	41	28	68
10	*K. phaffii*	*Xl*Nuc	Yeast	9 *	0	0
11	*K. phaffii*	*Xl*Nuc	*E. coli*	27 *	0	0
12	*K. phaffii*	*Xl*Nuc	Inscripta	30 *	0	0
13	*K. phaffii*	*SV*40	Yeast	12 *	0	0
14	*K. phaffii*	*SV*40	*E. coli*	31 *	14	45
15	*K. phaffii*	*SV*40	Inscripta	3 *	0	0
16	*K. phaffii*	*Pp*Set7	Yeast	24 *	0	0
17	*K. phaffii*	*Pp*Set7	*E. coli*	15 *	0	0
18	*K. phaffii*	*Pp*Set7	Inscripta	12 *	0	0
19	*H. sapiens* 1	*Xl*Nuc	*E. coli*	70	2	3
20	*H. sapiens* 1	*SV*40	*E. coli*	70	10	14
21	*H. sapiens* 1	*Pp*Set7	*E. coli*	70	63	90
22	*H. sapiens* 2	*Xl*Nuc	*E. coli*	70	10	14
23	*H. sapiens* 2	*SV*40	*E. coli*	70	0	0
24	*H. sapiens* 2	*Pp*Set7	*E. coli*	70	40	57
25	*H. sapiens* 3	*Xl*Nuc	*E. coli*	70	0	0
26	*H. sapiens* 3	*SV*40	*E. coli*	70	0	0
27	*H. sapiens* 3	*Pp*Set7	*E. coli*	70	57	81
28	*H. sapiens* 4	*Xl*Nuc	*E. coli*	70	5	7
29	*H. sapiens* 4	*SV*40	*E. coli*	70	0	0
30	*H. sapiens* 4	*Pp*Set7	*E. coli*	70	15	21

* All obtained transformants were screened.

**Table 4:** Effects of PAM sites on editing efficiency of MAD7 construct 8 at the *GUT1* locus.

#	PAM 5′ → 3′	gRNA	Total Number of Screened Clones	Number of Clones with Reduced Growth	Disruption Efficiency [%]
1	TTTA	1	42	12	29
2		2	42	0	0
3	TTTT	1	40	29	73
4		2	42	0	0
5	TTTC	1	42	35	83
6		2	42	21	50
7	TTTG	1	42	36	86
8		2	41	38	93
9	CTTA	1	42	1	2
10		2	42	0	0
11	CTTT	1	42	0	0
12		2	42	0	0
13	CTTC	1	42	0	0
14		2	42	0	0
15	CTTG	1	42	0	0
16		2	42	0	0

**Table 5:** Disruption efficiency of CRISPR-MAD7 construct 21 targeting DsRed and Sh ble.

Target	gRNA	Total Number of Screened Clones	Number of Disrupted Clones	Disruption Efficiency [%]
*DsRed*	1	42	32	76
	2	42	30	71
Sh ble	1	42	29	69
	2	42	32	76

**Table 6:** Summary of editing results using Cas9 and MAD7 endonucleases.

Nuclease	Frameshift Indels	In-Frame Indels	Edited/Targeted Genes
Cas9	142 (~84%)	27 (~16%)	169/259 (~65%)
MAD7	38 (~64%)	21 (~36%)	59/259 (~23%)

**Table 7:** Summary of preferable PAM sites for the CRISPR-MAD7 system.

	TTTT	TTTA	TTTG	TTTC
Number of gRNAs (% of total amount of designed gRNAs)	54 (21%)	37 (14%)	89 (34%)	80 (31%)
Number of editing events (% of total number of editing events)	10 (17%)	10 (17%)	17 (29%)	22 (36%)
Average editing efficiency (%)	19%	27%	19%	28%

**Table 8:** Summary of preferable PAM sites for the CRISPR-Cas9 system.

	AGG	TGG	CGG	GGG
Number of gRNAs (% of total amount of designed gRNAs)	84 (32%)	105 (40%)	37 (15%)	33 (13%)
Number of editing events (% of total number of editing events)	51 (30%)	65 (38%)	28 (17%)	25 (15%)
Average gene-editing efficiency (%)	61%	62%	76%	76%

**Table 9:** Summary of HDR-mediated editing efficiency.

Nuclease	BG10		BSYBG10 Δ*ku*70	
	NHEJ-Mediated Editing	HDR-Mediated Editing	NHEJ-Mediated Editing	HDR-Mediated Editing
Main group				
Cas9 (f)	7/7	1/7	0/7	7/7
MAD7 (n)	0/7	0/7	0/7	0/7
Control group				
Cas9 (f)	8/9	2/9	1/9	7/9
MAD7 (f)	8/9	4/9	0/9	4/9

f—functional; n—non-functional plasmids.

## Data Availability

All data supporting the findings of this study are available within the paper and within its Supplementary Materials published online.

## Supplementary Materials

The following are available online at https://www.mdpi.com/article/10.3390/jof10030197/s1. Supplementary File S1: S1: *DsRed* nucleotide sequence; S2: Sh ble nucleotide sequence; S3: sgRNA construct sequences; S4: CRISPR vector backbones nucleotide sequences; S5: MAD7 nucleotide sequences without NLS; S6: primers/ssDNA oligonucleotides; S7: gRNAs. Supplementary File S2: Table S1: list of targeted genes; Table S2: gRNAs sequences; Table S3: primers for gRNAs insertion; Table S4: donor DNA fragments; Table S5: primers for sequencing; Table S6: MAD7-Cas9 editing efficiency; Table S7: HDR editing efficiency. Supplementary File S3: DNA sequences of the target kinase genes and the alignments of Sanger sequencing results demonstrating the presence of indels.
