# Supplementary material for: Comparison of CRISPR-MAD7 and CRISPR-Cas9 for Gene Disruptions in Komagataella phaffii
Source: J Fungi (Basel). 2024 Mar 5;10(3):197. doi: 10.3390/jof10030197 (PMC10971144; doi:10.3390/jof10030197)
Supplement: Supplementary file 1 [file jof-10-00197-s001.zip › Supplementary_File_S1_v8.2.pdf]

Supplementary File S1

Comparison of CRISPR-MAD7 and CRISPR-Cas9 for gene disruptions in *Komagataella phaffii*

Kirill Smirnov[1], Florian Weiss[1], Anna-Maria Hatzl[1], Lukas Rieder[1], Kjeld Olesen[2], Sanne Jensen[2] and Anton Glieder [1]

[1] Christian Doppler Laboratory for Innovative Pichia pastoris host and vector systems, Institute of Molecular Biotechnology, Graz University of Technology, Graz, Austria;

[2] Novo Nordisk A/S, Novo Nordisk Park, DK 2760 Måløv, Denmark

Contents

S1: *DsRed* nucleotide sequence 2

S2: *Sh ble* nucleotide sequence 2

S3: sgRNA construct sequences 2

S4: CRISPR vector backbones nucleotide sequences (5'-3') 3

S5: MAD7 nucleotide sequences without NLS 7

S6: Primers/ssDNA Oligonucleotides 15

S7: gRNAs 17

S1: *DsRed* nucleotide sequence (5' - 3')

ATGGTTTCTAAGGGTGAGGAAGTTATCAAGGAGTTCATGCGTTTCAAGGTCAGAATGGAAGGT
TCTATGAACGGTCATGAGTTCGAGATTGAAGGAGAAGGTGAAGGAAGACCATATGAGGGTACT
CAAACCGCAAAGTTGAAGGTTACTAAAGGAGGTCCTTTACCATTCGCTTGGGATATCCTGTCTC
CACAATTCATGTATGGTTCTAAGGCATACGTTAAGCATCCTGCAGACATTCCTGACTACAAGAA
GTTGTCCTTTCCTGAGGGTTTCAAGTGGGAAAGAGTCATGAACTTCGAAGACGGTGGATTGGTG
ACTGTCACTCAAGACTCTTCCCTTCAAGACGGTACTTTGATCTACAAGGTCAAGATGCGTGGTA
CCAACTTCCCACCAGATGGTCCTGTTATGCAGAAAAAGACTATGGGATGGGAAGCTTCTACTGA
GAGATTGTATCCAAGAGATGGTGTTTTGAAGGGTGAGATTCACCAAGCTTTGAAGCTTAAAGA
TGGAGGTCACTACTTGGTTGAGTTCAAGACCATTTACATGGCTAAGAAACCAGTTCAACTTCCT
GGATACTATTACGTTGACACTAAGCTGGACATTACCTCTCACAACGAAGACTACACCATCGTTG
AGCAATACGAGAGATCCGAAGGTAGACACCACTTGTTCTTGTACGGTATGGACGAGCTTTATAA
GTAA

S2: Sh ble nucleotide sequence (5' - 3')

ATGGCTAAACTCACCTCTGCTGTTCCAGTCCTGACTGCTCGTGATGTTGCTGGTGCTGTTGAGTT
CTGGACTGATAGACTCGGTTTCTCCCGTGACTTCGTAGAGGACGACTTTGCCGGTGTTGTACGT
GACGACGTTACCCTGTTCATCTCCGCAGTTCAGGACCAGGTTGTGCCAGACAACACTCTGGCAT
GGGTATGGGTTCGTGGTCTGGACGAACTGTACGCTGAGTGGTCTGAGGTCGTGTCTACCAACTT
CCGTGATGCATCTGGTCCAGCTATGACCGAGATCGGTGAACAGCCCTGGGGTCGTGAGTTTGCA
CTGCGTGATCCAGCTGGTAACTGCGTGCATTTCGTCGCAGAAGAGCAGGACTAA

S3: sgRNA construct sequences (5' - 3')

With gRNA scaffold *E. coli* (CRISPR-MAD7 backbone):

AATTCCCTGATGAGTCCGTGAGGACGAAACGAGTAAGCTCGTCGGAATTTCTACTCTTGTAGAT
GCTAGCGGCCGGCATGGTCCCAGCCTCCTCGCTGGCGCCGGCTGGGCAACATGCTTCGGCATGGC
GAATGGGAC

With gRNA scaffold Yeasts (CRISPR-MAD7 backbone):

CCAGACCTGATGAGTCCGTGAGGACGAAACGAGTAAGCTCGTCGTCTGGCCCCAAATTCTAATT
TCTACTGTTGTAGATGCTAGCGGCCGGCATGGTCCCAGCCTCCTCGCTGGCGCCGGCTGGGCAAC
ATGCTTCGGCATGGCGAATGGGAC

With gRNA scaffold FAQ (CRISPR-MAD7 backbone):

TTTGACCTGATGAGTCCGTGAGGACGAAACGAGTAAGCTCGTCGTCAAAAGACCTTTTTAATTT
CTACTCTTGTAGATGCTAGCGGCCGGCATGGTCCCAGCCTCCTCGCTGGCGCCGGCTGGGCAACA
TGCTTCGGCATGGCGAATGGGAC

sgRNA cassette (CRISPR-Cas9 backbone):

AAAAAACTGATGAGTCCGTGAGGACGAAACGAGTAAGCTCGTCAAAAAACGAGACGACGAGTA
GGTAGCTCAACTAGTTGTCGAGGTCTGACCGTCTCTGTTTTAGAGCTAGAAATAGCAAGTTAAA
ATAAGGCTAGTCCGTTATCAACTTGAAAAAGTGGCACCGAGTCGGTGCTTTTGGCCGGCATGGT
CCCAGCCTCCTCGCTGGCGCCGGCTGGGCAACATGCTTCGGCATGGCGAATGGGAC

Reverse complementary sequence, Hammerhead ribozyme, gRNA scaffold, a site for gRNA insertion, HDV ribozyme

S4: CRISPR vector backbones nucleotide sequences (5'-3')

CRISPR-MAD7 backbone vector:

AGATCCAATTCCCGCTTTGACTGCCTGAAATCTCCATCGCCTACAATGATGACATTTGGATTTG
GTTGACTCATGTTGGTATTGTGAAATAGACGCAGATCGGGAACACTGAAAAATACACAGTTATT
ATTCATTTAAATGACCCTTGTGACTGACACTTTGGGAGTCCCTATTCTACTTAGTCTCATATCG
CATGAAACTTTTGATAAATTATTTTCTGATAGGAATTTTTCATCAGATATTATCATCGCGGCTT
ACGTAATAACAAAAAAAATTGATGGAGTCTATACTAGGCTAACATAAACTAAGTTATTAATTA
AACAAAACAAAACGTACTAGCATTACTGTCATATATAAGGGCTCCTAACTAAAACTGTAAAGAC
TTCCCGTCTCGAGTTAACCTTTTATCCGTTTGTTTCTCTTTGACCCTTCCTCTTCTTCAAGTTTT
CTCTTGAGATAGCGCTTATTCTGGATAAAGTCGAACCAATCTTTATTGCTGATTTTGAGTTTAT
CGCGCGAAAATTTACCATCTTCTTTCCAATTTTCGGTAATTTGTTTAATTTCATATAACCCTTT
TAATGCAATACAATACGCACCATTTGCATCGGCATCCTTAGGAAGTGCATCCCCCGCTTTCGCGC
TGTCATAAAAAATGTTATTTTCGTTCAGTACAGGTGAAATGAGACGATCGTAATCACGGTCCTC
CAGTTCAGACAAGGAGTTACGCATTTGTACGGTCAGGCGGAAGATTTCAAAGATGTGTTGCACA
ATTTCGTAGTCAATAATATCTTGCCGCAGATCATGGCCATCGCGCCAGTTAATATCGGTCATTT
CGAGTGTTTTCTCCATATCTTTGGTGATGTCTATAGTGTCGGACTCGTTGGAGAATCGTCCGTT
CACGAATCTTCTTTTGATGCGTACGCCGTAAGTGTAAACAGACCAGGAACTCTTAGACATAACA
GTATTCTGGGTAATAAAGTTGTTGTAGTCGAATGTGAAGCAGAACAGGTTCTTCTCGGAGTCGT
AGCGGATGCTGTCAAACTTCTTAATGAACTCTCTCTTGGCATCGACGGTCAAATCCTTGAATTT
AAAGATATTGACAAAGCCCGTTGTGGGATCGATCTTTGACGTGTAAGCAGCGGGGACGTAGAAA
ATGCAGCCGCATTGGTGACCGACGTTCTTCAGTTTGTCGGGGATATATGTGAGCTGGTATCCTT
TCAGCAGGCCGCCATTCTCCGTAATAGAAATGTCCTTGAAGACAAGATAATTGAGTTTGTTAAT
CAACATTGTCTCGAATTTCTGGTATACCTGCCTCTCGACCTTGAAGCGACCTTTCTTGAAGCCGT
AACTCAAGTCCTCCATGGCTATTATCGCGTTATACTTAATCACCATTTTACTTATTTCGTGAAT
GACCAGGGACAGATAGCCTTCCTTGATTTCCTTAATTTTCCCGATTTCCTTCCACTCCTTTCGGG
CGATCTGCCTTGCACCCTCCTGCTGTTTCAGCTTAATCTGATAGTCGTAGCCATTCACAATGTTA
AAAGATTTTTGCTCCACGATATTACCACAGGTGTCGATCACGGAAACGTAGATCAAGTTTCTCT
CACCCCGATCGATGCCGATCACGTGGAGGTCTTTCTCTTTGGCTATATACTGCAGAATCCTGTCG
TTGATAAATCCCGTCTTATTAGCCTTAAAGTTAATAGTAATCGGCATGTGCAGGAAATATTTGT
CATAAGTGTACCGATAGTCCTTAACAATGTTTGTTGCGGCTTCGTGATGGCCGACCACATTCTT
CAATTTAGCCGCCTCGTCGGAAAGCTCCTTGTCGCTTTTATCATTAAAGTACTTATAAAGTTCC
TGATAGATATTCTCTGGTATGTTTTTCCTGACAATTTGGATGTTACCAAATTGGTCCTTCTCCT
CGGCCTCGTATGTGCGATTGACGAGGATACTTCCCTTCTTGTGAATAATAGGGTTCTTGATTGA
GCTTTTTCGAAAGAAGATCTCGGCCTCCCCATTGAGTTTAAGCACAATGTCCTTGAGGTTTTCT
TCAGAGAAGAGATTCTTCAGGTACATGGTGTGCAGGTTATCGTTCCCGGTACTTTTCTTAGAGA
AGTCTTTGTTATAGATCTGGAAAAGGTAAAGCTGCCCCTTCTCCTGGAGGAGGTCGATGTCTTT
CTCACTGATGTATGTCCAATCAATTTTGTAGCCCTGCAGCTCCACTTCTCTGTAAAAGCCGCTAA
TATCCTCGTAAGTACTGGTATCGGAAAAATCGAAGCCGAAGTTTTTCCATTCGGGGTGAATAGC
GATGCAATTCTTAAAGTAGTCGATGAGGTCATGACAGAATGTTATATCGAAGTCTTTGGATGAC
TTAATGTGCTTGTTCTGCTTGTATCCTTCCAGGATGTAGGCGCTTGGTTTGTATGTCTCTACCCC
GGTTTTTGATGAGAGGAACACCTTAGGGATCATTTTGTTTGGACCTGGGAGCAGATTGTAGATC
ATCTTCTTGTAGTCGCCTTTGTTCTCGGAGGTATTCCCCTCAATGATCTTCTTGTCTGGCTTATT

TTTCGCATTGAAGATGCCCAGGTAGTACAGATTATCGCGCATAAGGATTATGGCATTGTTGGAG
TACTCCTTAGATTTACTCCAGCCGTCTGCGAGAGTAGGTATGCCAAAGTTCAGCTTGATCTTCT
TAGTAGAATATGGCTTTTGTGTCACGTAGTTCCTCACGAGATTATAGAGGGATATGACAGGGTA
AATCTCATCGTAGATTTCCTCCAGTTCGGCATAGAAGTTGTTATCTTTATCCACCAGTTCTTCT
GTCATGAACACAGAACACCAGTGGAAGGCATTCATGATCACATCCAACACATTCTTCAGCTCGC
TGGCTTTCAACTCTGACTCTACCAGGTGGATCTCAGGATTATACTTCAGTTCCTGTGCCTCAAA
ATTGTTCAGGATGTGTGAAATCTCGTGGATGTAAGTTTCGGCTTTGATATTATCGTCTGAACAC
AGCTTGTAGTTGCTAACCAGCTCGTTGATCTCAGTAATACTCTTCTGCAGATCATTCTTCACGG
CCTTTTTGACCTTGTCTGCCTTGGACTTGCCATTGCCAGGCAGGATATTGTTGTAGTGGATTTC
GAGAGCCGTGTTAATTGTCTCCCAGTCGCGATAGGTTTTCTGTGACACAGACTCGTAAAACTTG
GACACAATGTAGATTTTATCCAGGTTATAACCGTTATAATTGTCCCCGATCTTTCGCAGCCGCT
CCACAATGTGCTTAGAACTGATGTTGTCAAGAAACCCGTTAACACTCTGGTACACCTCCTCGTC
AGATTCAAATTTGTAGGGGACTTCGTAGCTGGTATCGGCGATACACAGGATCTGTTTGTGCAGC
TTCTGCAACTTGTACAAATTCTTATTCTCTTTGTTTTTCTGGCAATACAGATTCATAAAGCTGT
TCACCTTACCACAGATGTCGTTGTAAAAGCTTATACCCTCCTGGGTGATAAACTCGCCGTATTT
TTCATAAGAGTAGATCTCCTCCAGACTCATCTCCTTCAAGGAATCTTTCATGTCACCAGAGATT
TTGTTAATATCATCGTTGCTCAGGGATTTTACTATTCGCCTGTAGACGAGGGCGTTTGAAAAGA
AAATTTCGGCATTGTCATTCACGATCCGATGGCAACTACTGGAAGAGATATCGTCAGCTGAGAA
GCAGTTAGCGCGGTTTTTAAAGTAGTCTTTAAAGGACGTAGCGAATCTACTAAAAAGTTTGATG
ACCTGTGTCTTCTCCTCCTTTTCGCTGGCGCTGTAATTATTGTTATGTATCACGAACTCGGGAA
GTATATCGCTGATGAGTTTTGCGCTAAACATGTTTTTAAATCTGTCATCATTTGCAAATTTCTT
ATGTATGGCTTTTCTATACTCGGTCTGCTCTTTAATGAGGGTGTCTTTGTTATCGCCGTTCTTC
AGCTGAATCTCCATTTTCTCGAAAAGGCTGGTCCAATCAATATCATCAATGGAACTCAGGGTTT
CGCTAATGAACCCGCGGTAGTAATCGTCCATGATATCTTTCAGAATCTGGCGGTTTTCCCCTCT
AAGCTCATCTTCCTTGATTATACCGTTCTTCACAATAAATTGCTGAGTTGTCTCGGTAGGAATG
AGAGCGTTCCGCAGGGTCTTTTGGAGTGAGCTGATCCCTATAAAGTTCTGGAAATTGTTTGTAC
CGTTGTTCATCGTTTCGGAATTCTGTTGTAGTTTTAATATAGTTTGAGTATGAGATGGAACTCA
GAACGAAGGAATTATCACCAGTTTATATATTCTGAGGAAAGGGTGTGTCCTAAATTGGACAGTC
ACGATGGCAATAAACGCTCAGCCAATCAGAATGCAGGAGCCATAAATTGTTGTATTATTGCTGC
AAGATTTATGTGGGTTCACATTCCACTGAATGGTTTTCACTGTAGAATTGGTGTCCTAGTTGTT
ATGTTTCGAGATGTTTTCAAGAAAAACTAAAATGCACAAACTGACCAATAATGTGCCGTCGCGC
TTGGTACAAACGTCAGGATTGCCACCACTTTTTTCGCACTCTGGTACAAAAGTTCGCACTTCCC
ACTCGTATGTAACGAAAAACAGAGCAGTCTATCCAGAACGAGACAAATTAGCGCGTACTGTCCC
ATTCCATAAGGTATCATAGGAAACGAGAGTCCTCCCCCCATCACGTATATATAAACACACTGAT
ATCCCACATCCGCTTGTCACCAAACTAATACATCCAGTTCAAGTTACCTAAACAAATCAAAAAT
TCCCTGATGAGTCCGTGAGGACGAAACGAGTAAGCTCGTCGGAATTTCTACTCTTGTAGATGCT
AGCGGCCGGCATGGTCCCAGCCTCCTCGCTGGCGCCGGCTGGGCAACATGCTTCGGCATGGCGAA
TGGGACTCAAGAGGATGTCAGAATGCCATTTGCCTGAGAGATGCAGGCTTCATTTTTGATACTT
TTTTATTTGTAACCTATATAGTATAGGATTTTTTTTGTCATTTTGTTTCTTCTCGTACGAGCTT
GCTCCTGATCAGCCTATCTCGCAGCAGATGAATATCTTGTGGTAGGGGTTTGGGAAAATCATTC
GAGTTTGATGTTTTTCTTGGTATTTCCCACTCCTCTTCAGAGTACAGAAGATTAAGTGAGACCT
TCGTTTGTGCGGATCCTTCAGTAATGTCTTGTTTCTTTTGTTGCAGTGGTGAGCCATTTTGACT
TCGTGAAAGTTTCTTTAGAATAGTTGTTTCCAGAGGCCAAACATTCCACCCGTAGTAAAGTGCA
AGCGTAGGAAGACCAAGACTGGCATAAATCAGGTATAAGTGTCGAGCACTGGCAGGTGATCTTC
TGAAAGTTTCTACTAGCAGATAAGATCCAGTAGTCATGCATATGGCAACAATGTACCGTGTGGA
TCTAAGAACGCGTCCTACTAACCTTCGCATTCGTTGGTCCAGTTTGTTGTTATCGATCAACGTG
ACAAGGTTGTCGATTCCGCGTAAGCATGCATACCCAAGGACGCCTGTTGCAATTCCAAGTGAGC
CAGTTCCAACAATCTTTGTAATATTAGAGCACTTCATTGTGTTGCGCTTGAAAGTAAAATGCGA
ACAAATTAAGAGATAATCTCGAAACCGCGACTTCAAACGCCAATATGATGTGCGGCACACAATA
AGCGTTCATATCCGCTGGGTGACTTTCTCGCTTTAAAAAATTATCCGAAAAAATTTTCTAGAGT
GTTGTTACTTTATACTTCCGGCTCGTATAATACGACAAGGTGTAAGGAGGACTAAACCATGGCT
AAACTCACCTCTGCTGTTCCAGTCCTGACTGCTCGTGATGTTGCTGGTGCTGTTGAGTTCTGGAC

TGATAGACTCGGTTTCTCCCGTGACTTCGTAGAGGACGACTTTGCCGGTGTTGTACGTGACGAC
GTTACCCTGTTCATCTCCGCAGTTCAGGACCAGGTTGTGCCAGACAACACTCTGGCATGGGTAT
GGGTTCGTGGTCTGGACGAACTGTACGCTGAGTGGTCTGAGGTCGTGTCTACCAACTTCCGTGA
TGCATCTGGTCCAGCTATGACCGAGATCGGTGAACAGCCCTGGGGTCGTGAGTTTGCACTGCGT
GATCCAGCTGGTAACTGCGTGCATTTCGTCGCAGAAGAGCAGGACTAACAATTGACACCTTACG
ATTATTTAGAGAGTATTTATTAGTTTTATTGTATGTATACGGATGTTTTATTATCTATTTATGC
CCTTATATTCTGTAACTATCCAAAAGTCCTATCTTATCAAGCCAGCAATCTATGTCCGCGAACG
TCAACTAAAAATAAGCTTTTTATGCTCTTCTCTCTTTTTTTCCCTTCGGTATAATTATACCTTGC
ATCCACAGATTCTCCTGCCAAATTTTGCATAATCCTTTACAACATGGCTATATGGGAGCACTTA
GCGCCCTCCAAAACCCATATTGCCTACGCATGTATAGGTGTTTTTTCCACAATATTTTCTCTGTG
CTCTCTTTTTATTAAAGAGAAGCTCTATATCGGAGAAGCTTCTGTGGCCGTTATATTCGGCCTT
ATCGTGGGACCACATTGCCTGAATTGGTTTGCCCCGGAAGATTGGGGAAACTTGGATCTGATTA
CCTTAGCTGCATCGAGATAAGCTGGGGGAACATTCGCGAAAATGAAACAAGTCGGCTGTTATAG
TATATTTATTATAATATTGAAAGATCTCAAAAGACTACTTATTTTTGAATGAACCAAGTATGA
AATCAACCTATTTGGGGTTGACCAAAATAAGTAAATATTAATTGTCGAGGTACCACTGAGCGTC
AGACCCCGTAGAAAAGATCAAAGGATCTTCTTGAGATCCTTTTTTTCTGCGCGTAATCTGCTGC
TTGCAAACAAAAAAACCACCGCTACCAGCGGTGGTTTGTTTGTCGGATCAAGAGCTACCAACTC
TTTTTCCGAAGGTAACTGGCTTCAGCAGAGCGCAGATACCAAATACTGTTCTTCTAGTGTAGCC
GTAGTTAGGCCACCACTTCAAGAACTCTGTAGCACCGCCTACATACCTCGCTCTGCTAATCCTGT
TACCAGTGGCTGCTGCCAGTGGCGATAAGTCGTGTCTTACCGGGTTGGACTCAAGACGATAGTT
ACCGGATAAGGCGCAGCGGTCGGGCTGAACGGGGGGTTCGTGCACACAGCCCAGCTTGGAGCGA
ACGACCTACACCGAACTGAGATACCTACAGCGTGAGCTATGAGAAAGCGCCACGCTTCCCGAAG
GGAGAAAGGCGGACAGGTATCCGGTAAGCGGCAGGGTCGGAACAGGAGAGCGCACGAGGGAGC
TTCCAGGGGGAAACGCCTGGTATCTTTATAGTCCTGTCGGGTTTCGCCACCTCTGACTTGAGCG
TCGATTTTTGTGATGCTCGTCAGGGGGGCGGAGCCTATGGAAAAACGCCAGCAACGCGGCCTTT
TTACGGTTCCTGGCCTTTTGCTGGCCTTTTGCTCACATGTTCTTTCCTGCGGTACCC

CRISPR-Cas9 backbone vector:

TCAAGAGGATGTCAGAATGCCATTTGCCTGAGAGATGCAGGCTTCATTTTTGATACTTTTTTAT
TTGTAACCTATATAGTATAGGATTTTTTTTGTCATTTTGTTTCTTCTCGTACGAGCTTGCTCCT
GATCAGCCTATCTCGCAGCAGATGAATATCTTGTGGTAGGGGTTTGGGAAAATCATTCGAGTTT
GATGTTTTTCTTGGTATTTCCCACTCCTCTTCAGAGTACAGAAGATTAAGTGAGACCTTCGTTT
GTGCGGATCCTTCAGTAATGTCTTGTTTCTTTTGTTGCAGTGGTGAGCCATTTTGACTTCGTGA
AAGTTTCTTTAGAATAGTTGTTTCCAGAGGCCAAACATTCCACCCGTAGTAAAGTGCAAGCGTA
GGAAGACCAAGACTGGCATAAATCAGGTATAAGTGTCGAGCACTGGCAGGTGATCTTCTGAAAG
TTTCTACTAGCAGATAAGATCCAGTAGTCATGCATATGGCAACAATGTACCGTGTGGATCTAAG
AACGCGTCCTACTAACCTTCGCATTCGTTGGTCCAGTTTGTTGTTATCGATCAACGTGACAAGG
TTGTCGATTCCGCGTAAGCATGCATACCCAAGGACGCCTGTTGCAATTCCAAGTGAGCCAGTTC
CAACAATCTTTGTAATATTAGAGCACTTCATTGTGTTGCGCTTGAAAGTAAAATGCGAACAAAT
TAAGAGATAATCTCGAAACCGCGACTTCAAACGCCAATATGATGTGCGGCACACAATAAGCGTT
CATATCCGCTGGGTGACTTTCTCGCTTTAAAAAATTATCCGAAAAAATTTTCTAGAGTGTTGTT
ACTTTATACTTCCGGCTCGTATAATACGACAAGGTGTAAGGAGGACTAAACCATGGCTAAACTC
ACCTCTGCTGTTCCAGTCCTGACTGCTCGTGATGTTGCTGGTGCTGTTGAGTTCTGGACTGATA
GACTCGGTTTCTCCCGTGACTTCGTAGAGGACGACTTTGCCGGTGTTGTACGTGACGACGTTAC
CCTGTTCATCTCCGCAGTTCAGGACCAGGTTGTGCCAGACAACACTCTGGCATGGGTATGGGTT
CGTGGTCTGGACGAACTGTACGCTGAGTGGTCTGAGGTCGTGTCTACCAACTTCCGTGATGCAT
CTGGTCCAGCTATGACCGAGATCGGTGAACAGCCCTGGGGTCGTGAGTTTGCACTGCGTGATCC
AGCTGGTAACTGCGTGCATTTCGTCGCAGAAGAGCAGGACTAACAATTGACACCTTACGATTAT
TTAGAGAGTATTTATTAGTTTTATTGTATGTATACGGATGTTTTATTATCTATTTATGCCCTTA
TATTCTGTAACTATCCAAAAGTCCTATCTTATCAAGCCAGCAATCTATGTCCGCGAACGTCAAC
TAAAAATAAGCTTTTTATGCTCTTCTCTCTTTTTTTCCCTTCGGTATAATTATACCTTGCATCCA

CAGATTCTCCTGCCAAATTTTGCATAATCCTTTACAACATGGCTATATGGGAGCACTTAGCGCC
CTCCAAAACCCATATTGCCTACGCATGTATAGGTGTTTTTTCCACAATATTTTCTCTGTGCTCTC
TTTTTATTAAAGAGAAGCTCTATATCGGAGAAGCTTCTGTGGCCGTTATATTCGGCCTTATCGT
GGGACCACATTGCCTGAATTGGTTTGCCCCGGAAGATTGGGGAAACTTGGATCTGATTACCTTA
GCTGCATCGAGATAAGCTGGGGGAACATTCGCGAAAATGAAACAAGTCGGCTGTTATAGTATAT
TTATTATAATATTGAAAGATCTCAAAAGACTACTTATTTTTGAATGAACCAAGTATGAAATCA
ACCTATTTGGGGTTGACCAAAATAAGTAAATATTAATTGTCGAGGTACCACTGAGCGTCAGACC
CCGTAGAAAAGATCAAAGGATCTTCTTGAGATCCTTTTTTTCTGCGCGTAATCTGCTGCTTGCA
AACAAAAAAACCACCGCTACCAGCGGTGGTTTGTTTGTCGGATCAAGAGCTACCAACTCTTTTT
CCGAAGGTAACTGGCTTCAGCAGAGCGCAGATACCAAATACTGTTCTTCTAGTGTAGCCGTAGT
TAGGCCACCACTTCAAGAACTCTGTAGCACCGCCTACATACCTCGCTCTGCTAATCCTGTTACCA
GTGGCTGCTGCCAGTGGCGATAAGTCGTGTCTTACCGGGTTGGACTCAAGACGATAGTTACCGG
ATAAGGCGCAGCGGTCGGGCTGAACGGGGGGTTCGTGCACACAGCCCAGCTTGGAGCGAACGAC
CTACACCGAACTGAGATACCTACAGCGTGAGCTATGAGAAAGCGCCACGCTTCCCGAAGGGAGA
AAGGCGGACAGGTATCCGGTAAGCGGCAGGGTCGGAACAGGAGAGCGCACGAGGGAGCTTCCAG
GGGGAAACGCCTGGTATCTTTATAGTCCTGTCGGGTTTCGCCACCTCTGACTTGAGCGTCGATT
TTTGTGATGCTCGTCAGGGGGGCGGAGCCTATGGAAAAACGCCAGCAACGCGGCCTTTTTACGG
TTCCTGGCCTTTTGCTGGCCTTTTGCTCACATGTTCTTTCCTGCGGTACCCAGATCCAATTCCCG
CTTTGACTGCCTGAAATCTCCATCGCCTACAATGATGACATTTGGATTTGGTTGACTCATGTTG
GTATTGTGAAATAGACGCAGATCGGGAACACTGAAAAATACACAGTTATTATTCATTTAAATG
ACCCTTGTGACTGACACTTTGGGAGTCCCTATTCTACTTAGTCTCATATCGCATGAAACTTTTG
ATAAATTATTTTCTGATAGGAATTTTTCATCAGATATTATCATCGCGGCTTACGTAATAACAAA
AAAAATTGATGGAGTCTATACTAGGCTAACATAAACTAAGTTATTAATTAAACAAAACAAAAC
GTACTAGCATTACTGTCATATATAAGGGCTCCTAACTAAAACTGTAAAGACTTCCCGTCTAGAG
TTAAACTTTTCTTTTCTTCTTTGGGTCTCCACCGAGCTGAGAGAGGTCGATTCTTGTTTCATAG
AGCCCCGTAATTGACTGATGAATCAGTGTGGCGTCCAGGACCTCCTTTGTAGAGGTGTACCGCT
TTCTGTCTATGGTGGTGTCGAAGTACTTGAAGGCTGCAGGCGCGCCCAAGTTGGTCAGAGTAAA
CAAGTGGATAATGTTTTCTGCCTGCTCCCTGATGGGCTTATCCCTGTGCTTATTGTAAGCAGAA
AGCACCTTATCGAGGTTAGCGTCGGCGAGGATCACTCTTTTGGAGAATTCGCTTATTTGCTCGA
TGATCTCATCAAGGTAGTGTTTGTGTTGTTCCACGAACAGCTGCTTCTGCTCATTATCTTCGGG
AGACCCTTTGAGCTTTTCATAGTGGCTGGCCAGATACAAGAAATTAACGTATTTAGAGGGCAGT
GCCAGCTCGTTACCTTTCTGCAGCTCGCCCGCACTAGCGAGCATTCGTTTCCGGCCGTTTTCAAG
CTCAAAGAGAGAGTACTTGGGAAGCTTAATGATGAGGTCTTTTTTGACCTCTTTATATCCTTTC
GCCTCGAGAAAGTCGATGGGGTTTTTTTCGAAGCTTGATCGCTCCATGATTGTGATGCCCAGCA
GTTCCTTGACGCTTTTGAGTTTTTTAGACTTCCCTTTCTCCACTTTGGCCACAACCAGTACACTG
TAAGCGACTGTAGGAGAATCGAATCCGCCGTATTTCTTGGGGTCCCAATCTTTTTTGCGTGCGA
TCAGCTTGTCGCTGTTCCTTTTCGGGAGGATACTTTCCTTGGAGAAGCCTCCGGTCTGTACTTCG
GTCTTTTTAACGATGTTCACCTGCGGCATGGACAGGACCTTCCGGACTGTCGCGAAATCCCTACC
CTTGTCCCACACGATTTCTCCTGTTTCTCCGTTTGTTTCGATAAGTGGTCGCTTCCGAATCTCTC
CATTGGCCAGTGTAATCTCGGTCTTGAAAAAATTCATAATATTGCTGTAAAAGAAGTACTTAGC
GGTGGCCTTGCCTATTTCCTGCTCAGACTTTGCGATCATTTTCCTAACATCGTACACTTTATAGT
CTCCGTAAACAAATTCAGATTCAAGCTTGGGATATTTTTTGATAAGTGCAGTGCCTACCACTGC
ATTCAGGTAGGCATCATGCGCATGGTGGTAATTGTTGATCTCTCTCACCTTATAAAACTGAAAG
TCCTTTCTGAAATCTGAGACCAGCTTAGACTTCAGAGTAATAACTTTCACCTCTCGAATCAGTT
TGTCATTTTCATCGTACTTGGTGTTCATGCGTGAATCGAGAATTTGGGCCACGTGCTTGGTGAT
CTGGCGTGTCTCAACAAGCTGCCTTTTGATGAAGCCGGCTTTATCCAACTCAGACAGGCCACCTC
GTTCAGCCTTAGTCAGATTATCGAACTTCCGTTGTGTGATCAGTTTGGCGTTCAGCAGCTGCCG
CCAATAATTTTTCATTTTCTTGACAACTTCTTCTGAGGGGACGTTATCACTCTTCCCTCTATTTT
TATCGGATCTTGTCAACACTTTATTATCAATAGAATCATCTTTGAGAAAAGACTGGGGCACGAT
ATGATCCACGTCGTAGTCGGAGAGCCGATTGATGTCCAGTTCCTGATCCACGTACATGTCCCTG
CCGTTCTGCAGGTAGTACAGGTAGAGCTTCTCATTCTGAAGCTGGGTGTTTTCAACTGGGTGTT
CCTTAAGGATTTGGGACCCCAGTTCTTTTATACCCTCTTCAATCCTCTTCATCCTTTCCCTACTG

TTCTTCTGTCCCTTCTGGGTAGTTTGGTTCTCTCGGGCCATCTCGATAACGATATTCTCGGGCTT
ATGCCTTCCCATTACTTTGACGAGTTCATCCACGACCTTAACGGTCTGCAGTATTCCCTTTTTGA
TAGCTGGGCTACCTGCAAGATTAGCGATGTGCTCGTGAAGACTGTCCCCCTGGCCAGAAACTTG
TGCTTTCTGGATGTCCTCCTTAAAGGTGAGAGAGTCATCATGGATCAACTGCATGAAGTTCCGG
TTGGCAAATCCATCGGACTTAAGAAAATCCAGGATTGTCTTTCCACTCTGCTTGTCTCGGATCC
CATTGATCAGTTTTCTTGACAGCCGCCCCCATCCTGTATATCGGCGCCTCTTGAGCTGTTTCATG
ACTTTGTCGTCGAAGAGATGAGCGTAAGTTTTCAAGCGTTCTTCAATCATCTCCCTATCTTCAA
ACAACGTAAGGGTGAGGACAATGTCCTCAAGAATGTCCTCGTTCTCCTCATTGTCCAGGAAGTC
CTTGTCTTTAATGATTTTCAGGAGATCGTGATACGTTCCCAGGGATGCGTTGAAGCGATCCTCC
ACTCCGCTGATTTCAACAGAGTCGAAACATTCAATCTTTTTGAAATAGTCTTCTTTGAGCTGTT
TCACGGTAACTTTCCGGTTCGTCTTGAAGAGGAGGTCCACGATAGCTTTCTTCTGCTCTCCAGAC
AGGAATGCTGGCTTTCTCATCCCTTCTGTGACGTATTTGACCTTGGTGAGCTCGTTATAAACTG
TGAAGTACTCGTACAGCAGAGAGTGTTTAGGAAGCACCTTTTCGTTAGGCAGATTTTTATCAAA
GTTAGTCATCCTTTCGATGAAGGACTGGGCAGAGGCCCCCTTATCCACGACTTCCTCGAAGTTCC
AGGGAGTGATGGTCTCTTCTGATTTGCGAGTCATCCACGCGAATCTGGAATTTCCCCGGGCGAG
GGGGCCTACATAGTAGGGTATCCGAAATGTGAGGATTTTCTCAATCTTTTCCCTGTTATCTTTC
AAAAAGGGGTAGAAATCCTCTTGCCGCCTGAGGATAGCGTGCAGTTCGCCCAGGTGAATCTGGT
GGGGGATGCTTCCATTGTCGAAAGTGCGCTGTTTGCGCAACAGATCTTCTCTGTTAAGCTTTAC
CAGCAGCTCCTCGGTGCCGTCCATTTTTTCCAAGATGGGCTTAATAAATTTGTAAAATTCCTCC
TGGCTTGCTCCGCCGTCAATGTATCCGGCGTAGCCATTTTTAGACTGATCGAAGAAAATTTCCT
TGTACTTCTCAGGCAGTTGCTGTCTGACAAGGGCCTTCAGCAAAGTCAAGTCTTGGTGGTGCTC
ATCATAGCGCTTGATCATACTAGCGCTCAGCGGAGCTTTGGTGATCTCCGTGTTCACTCGCAGA
ATATCACTCAGCAGAATGGCGTCTGACAGGTTCTTTGCCGCCAAAAAAAGGTCTGCGTACTGGT
CGCCGATCTGGGCCAGCAGATTGTCGAGATCATCATCGTAGGTGTCTTTGCTCAGTTGAAGCTT
GGCATCTTCGGCCAGGTCGAAGTTAGATTTAAAGTTGGGGGTCAGCCCGAGTGACAGGGCGATA
AGATTACCAAACAGGCCGTTCTTCTTCTCCCCAGGGAGCTGTGCGATGAGGTTTTCGAGCCGCC
GGGATTTGGACAGCCTAGCGCTCAGGATTGCTTTGGCGTCAACTCCGGATGCGTTGATCGGGTT
CTCTTCGAAAAGCTGATTGTAAGTCTGAACCAGTTGGATAAAGAGTTTGTCGACATCGCTGTTG
TCTGGGTTCAGGTCCCCCTCGATGAGGAAGTGTCCCCGAAATTTGATCATATGCGCCAGCGCGA
GATAGATCAACCGCAAGTCAGCCTTATCAGTACTGTCTACAAGCTTCTTCCTCAGATGATATAT
GGTTGGGTACTTTTCATGGTACGCCACCTCGTCCACGATATTGCCAAAGATTGGGTGGCGCTCG
TGCTTTTTATCCTCCTCCACCAAAAAGGACTCCTCCAGCCTATGGAAGAAAGAGTCATCCACCTT
AGCCATCTCATTACTAAAGATCTCCTGCAGGTAGCAGATCCGATTCTTTCTGCGGGTATATCTG
CGCCGTGCTGTTCTTTTGAGCCGCGTGGCTTCGGCCGTTTCCCCGGAGTCGAACAGGAGGGCGCC
AATGAGGTTCTTCTTTATGCTGTGGCGATCGGTATTGCCCAGAACTTTGAATTTTTTGCTCGGC
ACCTTGTACTCGTCCGTAATGACGGCCCAGCCGACGCTGTTTGTGCCGATATCGAGCCCAATGGA
GTACTTCTTGTCCATCGTTTCGGAATTCTGTTGTAGTTTTAATATAGTTTGAGTATGAGATGGA
ACTCAGAACGAAGGAATTATCACCAGTTTATATATTCTGAGGAAAGGGTGTGTCCTAAATTGGA
CAGTCACGATGGCAATAAACGCTCAGCCAATCAGAATGCAGGAGCCATAAATTGTTGTATTATT
GCTGCAAGATTTATGTGGGTTCACATTCCACTGAATGGTTTTCACTGTAGAATTGGTGTCCTAG
TTGTTATGTTTCGAGATGTTTTCAAGAAAAACTAAAATGCACAAACTGACCAATAATGTGCCGT
CGCGCTTGGTACAAACGTCAGGATTGCCACCACTTTTTTCGCACTCTGGTACAAAAGTTCGCAC
TTCCCACTCGTATGTAACGAAAAACAGAGCAGTCTATCCAGAACGAGACAAATTAGCGCGTACT
GTCCCATTCCATAAGGTATCATAGGAAACGAGAGTCCTCCCCCCATCACGTATATATAAACACA
CTGATATCCCACATCCGCTTGTCACCAAACTAATACATCCAGTTCAAGTTACCTAAACAAATCA
AAaaaaaaCTGATGAGTCCGTGAGGACGAAACGAGTAAGCTCGTCAAAAAACGAGACGACGAGTA
GGTAGCTCAACTAGTTGTCGAGGTCTGACCGTCTCTGTTTTAGAGCTAGAAATAGCAAGTTAAA
ATAAGGCTAGTCCGTTATCAACTTGAAAAAGTGGCACCGAGTCGGTGCTTTTGGCCGGCATGGT
CCCAGCCTCCTCGCTGGCGCCGGCTGGGCAACATGCTTCGGCATGGCGAATGGGAC

S5: *MAD7* nucleotide sequences (5’ - 3’) without NLS:

PpMAD7:

ATGAACAACGGAACTAACAACTTTCAAAACTTTATTGGAATTTCTTCTTTGCAAAAGACTCTTA
GAAACGCTTTAATCCCAACCGAAACCACCCAGCAATTTATCGTTAAGAACGGTATCATCAAGGA
GGACGAGCTGAGAGGTGAAAACAGACAAATCCTGAAGGACATCATGGACGACTACTACAGAGG
ATTCATTTCCGAGACTTTGTCTTCCATCGATGACATTGACTGGACTTCACTGTTCGAGAAAATG
GAAATCCAATTGAAGAACGGTGACAACAAGGATACTTTGATTAAGGAACAGACCGAATACAGA
AAGGCTATCCATAAGAAATTTGCTAACGACGATAGATTCAAGAATATGTTCTCCGCTAAGTTGA
TCTCTGACATTTTGCCAGAGTTTGTGATTCACAACAACAATTACTCTGCTTCTGAGAAGGAAGA
GAAGACCCAAGTCATCAAATTGTTCTCACGTTTTGCAACTTCTTTTAAGGACTACTTTAAAAAC
AGAGCCAACTGTTTTTCTGCTGATGATATTTCATCTTCCTCTTGTCACAGAATCGTGAACGATA
ACGCCGAAATCTTCTTCTCCAATGCTTTGGTCTACCGTAGAATCGTCAAATCTTTGTCTAACGA
TGATATCAATAAAATCTCCGGAGACATGAAGGATTCTTTGAAAGAGATGTCCTTGGAGGAGATC
TACTCCTATGAGAAGTATGGTGAATTCATTACTCAAGAGGGTATCTCTTTCTACAACGACATCT
GTGGTAAGGTTAATTCATTCATGAATTTGTACTGCCAAAAGAACAAGGAGAACAAGAACCTTT
ATAAGCTTCAAAAGTTGCACAAGCAAATCTTATGTATCGCCGACACTTCTTATGAGGTTCCATA
TAAGTTTGAGTCCGACGAGGAGGTCTACCAGTCCGTCAACGGTTTCCTGGACAACATCTCTTCT
AAGCATATTGTTGAAAGACTTAGAAAAATTGGAGATAACTATAACGGATACAACTTGGACAAG
ATTTACATTGTCTCTAAGTTCTACGAATCTGTTTCTCAGAAGACTTACAGAGACTGGGAAACTA
TTAATACCGCTTTGGAGATCCACTACAACAACATCCTTCCAGGTAACGGTAAGTCTAAGGCCGA
CAAGGTTAAGAAAGCCGTTAAGAACGACTTACAAAAATCCATCACTGAGATTAATGAGTTGGT
GTCTAACTATAAGTTGTGCTCTGATGACAACATCAAAGCTGAGACTTACATTCACGAAATTTCT
CACATCTTAAACAACTTCGAGGCCCAAGAGTTGAAGTACAACCCAGAGATCCACCTTGTTGAAT
CCGAACTTAAAGCCTCCGAGCTGAAGAACGTCTTGGACGTTATTATGAACGCTTTTCACTGGTG
TTCCGTGTTCATGACTGAAGAGCTTGTGGACAAGGATAATAACTTTTACGCAGAACTGGAGGAA
ATCTACGACGAGATTTACCCTGTTATTTCTCTGTACAATCTTGTTAGAAACTACGTCACCCAAA
AGCCTTACTCTACCAAGAAAATCAAGCTTAACTTTGGAATCCCTACTCTTGCCGACGGTTGGTC
CAAATCTAAGGAATACTCCAATAATGCTATCATTTTAATGAGAGATAACTTGTATTACTTGGGT
ATTTTCAACGCTAAGAACAAGCCTGACAAGAAGATTATTGAAGGTAACACCTCCGAGAACAAGG
GAGATTACAAGAAGATGATCTACAACCTGCTTCCTGGTCCTAACAAGATGATTCCAAAGGTCTT
CTTATCCTCTAAGACTGGAGTTGAGACTTACAAGCCATCTGCTTATATCCTGGAGGGTTACAAG
CAAAACAAACACATCAAGTCTTCCAAGGATTTTGACATTACCTTTTGTCACGATCTGATCGACT
ACTTCAAGAACTGTATCGCCATCCACCCAGAGTGGAAGAACTTCGGTTTCGACTTCTCTGACAC
TTCCACTTACGAGGATATTTCCGGTTTCTATAGAGAGGTGGAATTGCAAGGATACAAAATTGAC
TGGACCTACATCTCCGAAAAGGACATTGACCTTTTGCAAGAAAAAGGTCAATTGTACCTGTTCC
AAATTTACAACAAGGACTTTTCTAAGAAATCTACCGGTAACGATAACTTACATACCATGTACTT
GAAAAACCTTTTCTCTGAGGAAAACTTGAAGGACATCGTGTTGAAGTTGAACGGTGAGGCAGA
AATCTTCTTTCGTAAGTCTTCTATCAAGAACCCTATTATTCACAAGAAAGGATCAATCTTGGTT
AACAGAACCTACGAAGCCGAAGAGAAAGACCAATTCGGAAACATTCAAATTGTCAGAAAGAAC
ATTCCTGAAAACATTTATCAAGAGTTGTACAAGTACTTCAATGACAAGTCCGACAAGGAGTTGT
CTGATGAAGCTGCCAAGCTTAAGAACGTGGTGGGTCACCACGAAGCTGCTACCAACATTGTTAA
GGACTACAGATACACCTACGACAAGTACTTTTTGCACATGCCAATTACCATTAACTTCAAGGCC
AACAAGACCGGATTCATCAATGATAGAATTTTGCAATACATTGCCAAGGAGAAGGACTTGCACG
TGATTGGTATCGATAGAGGTGAGAGAAACTTGATTTACGTGTCAGTTATTGATACTTGCGGTAA
CATTGTCGAACAAAAGTCTTTTAACATCGTCAACGGTTATGACTACCAAATCAAGCTGAAGCAA
CAGGAGGGAGCTAGACAGATTGCAAGAAAGGAATGGAAGGAGATTGGTAAGATCAAAGAGATC
AAAGAGGGTTACCTTTCATTGGTTATTCATGAGATTTCTAAGATGGTGATTAAGTACAATGCTA
TCATCGCTATGGAGGACCTGTCTTACGGTTTCAAGAAAGGAAGATTCAAGGTCGAGAGACAAGT
TTACCAAAAATTTGAAACCATGTTGATTAACAAGCTGAACTACTTAGTTTTTAAAGACATTTCT
ATCACCGAGAATGGTGGTCTTTTGAAGGGTTACCAATTGACCTACATTCCTGACAAGTTGAAGA
ACGTCGGTCACCAATGCGGTTGTATTTTCTATGTTCCTGCCGCTTACACCTCCAAGATTGACCCA
ACTACCGGTTTCGTCAATATCTTCAAGTTCAAGGACCTTACTGTCGACGCTAAAAGAGAATTCA

TTAAGAAGTTCGACTCTATTAGATACGACTCAGAAAAGAATTTATTCTGTTTCACTTTCGATTA
CAACAACTTTATCACTCAGAACACTGTTATGTCTAAGTCCTCTTGGTCAGTTTACACTTACGGT
GTGCGTATTAAGAGAAGATTCGTTAATGGTAGATTCTCTAACGAATCTGACACTATTGATATTA
CCAAGGACATGGAGAAGACTTTAGAGATGACCGACATTAACTGGAGAGACGGTCACGATTTGAG
ACAAGATATTATTGATTACGAGATCGTGCAACACATTTTCGAAATCTTCAGATTGACTGTTCAA
ATGAGAAACTCCTTGTCTGAATTGGAAGATCGTGATTATGACAGACTTATTTCTCCAGTTTTAA
ATGAAAACAACATTTTCTATGATTCCGCTAAAGCTGGTGACGCATTGCCTAAAGACGCTGATGC
TAACGGTGCATACTGTATCGCTCTGAAAGGTTTGTACGAAATCAAGCAAATTACCGAGAACTGG
AAGGAGGACGGTAAGTTCTCAAGAGACAAGTTAAAGATTTCAAACAAAGATTGGTTTGATTTC
ATTCAAAACAAAAGATACTTGTAA

EcMAD7:

ATGAACAACGGCACAAATAATTTTCAGAACTTCATCGGGATCTCAAGTTTGCAGAAAACGCTGC
GCAATGCTCTGATCCCCACGGAAACCACGCAACAGTTCATCGTCAAGAACGGAATAATTAAAGA
AGATGAGTTACGTGGCGAGAACCGCCAGATTCTGAAAGATATCATGGATGACTACTACCGCGGA
TTCATCTCTGAGACTCTGAGTTCTATTGATGACATAGATTGGACTAGCCTGTTCGAAAAAATGG
AAATTCAGCTGAAAAATGGTGATAATAAAGATACCTTAATTAAGGAACAGACAGAGTATCGGA
AAGCAATCCATAAAAAATTTGCGAACGACGATCGGTTTAAGAACATGTTTAGCGCCAAACTGAT
TAGTGACATATTACCTGAATTTGTCATCCACAACAATAATTATTCGGCATCAGAGAAAGAGGAA
AAAACCCAGGTGATAAAATTGTTTTCGCGCTTTGCGACTAGCTTTAAAGATTACTTCAAGAACC
GTGCAAATTGCTTTTCAGCGGACGATATTTCATCAAGCAGCTGCCATCGCATCGTCAACGACAA
TGCAGAGATATTCTTTTCAAATGCGCTGGTCTACCGCCGGATCGTAAAATCGCTGAGCAATGAC
GATATCAACAAAATTTCGGGCGATATGAAAGATTCATTAAAAGAAATGAGTCTGGAAGAAATA
TATTCTTACGAGAAGTATGGGGAATTTATTACCCAGGAAGGCATTAGCTTCTATAATGATATCT
GTGGGAAAGTGAATTCTTTTATGAACCTGTATTGTCAGAAAAATAAAGAAAACAAAAATTTAT
ACAAACTTCAGAAACTTCACAAACAGATTCTATGCATTGCGGACACTAGCTATGAGGTCCCGTA
TAAATTTGAAAGTGACGAGGAAGTGTACCAATCAGTTAACGGCTTCCTTGATAACATTAGCAGC
AAACATATAGTCGAAAGATTACGCAAAATCGGCGATAACTATAACGGCTACAACCTGGATAAA
ATTTATATCGTGTCCAAATTTTACGAGAGCGTTAGCCAAAAAACCTACCGCGACTGGGAAACAA
TTAATACCGCCCTCGAAATTCATTACAATAATATCTTGCCGGGTAACGGTAAAAGTAAAGCCGA
CAAAGTAAAAAAAGCGGTTAAGAATGATTTACAGAAATCCATCACCGAAATAAATGAACTAGT
GTCAAACTATAAGCTGTGCAGTGACGACAACATCAAAGCGGAGACTTATATACATGAGATTAGC
CATATCTTGAATAACTTTGAAGCACAGGAATTGAAATACAATCCGGAAATTCACCTAGTTGAAT
CCGAGCTCAAAGCGAGTGAGCTTAAAAACGTGCTGGACGTGATCATGAATGCGTTTCATTGGTG
TTCGGTTTTTATGACTGAGGAACTTGTTGATAAAGACAACAATTTTTATGCGGAACTGGAGGAG
ATTTACGATGAAATTTATCCAGTAATTAGTCTGTACAACCTGGTTCGTAACTACGTTACCCAGA
AACCGTACAGCACGAAAAAGATTAAATTGAACTTTGGAATACCGACGTTAGCAGACGGTTGGTC
AAAGTCCAAAGAGTATTCTAATAACGCTATCATACTGATGCGCGACAATCTGTATTATCTGGGC
ATCTTTAATGCGAAGAATAAACCGGACAAGAAGATTATCGAGGGTAATACGTCAGAAAATAAG
GGTGACTACAAAAAGATGATTTATAATTTGCTCCCGGGTCCCAACAAAATGATCCCGAAAGTTT
TCTTGAGCAGCAAGACGGGGGTGGAAACGTATAAACCGAGCGCCTATATCCTAGAGGGGTATAA
ACAGAATAAACATATCAAGTCTTCAAAAGACTTTGATATCACTTTCTGTCATGATCTGATCGAC
TACTTCAAAAACTGTATTGCAATTCATCCCGAGTGGAAAAACTTCGGTTTTGATTTTAGCGACA
CCAGTACTTATGAAGACATTTCCGGGTTTTATCGTGAGGTAGAGTTACAAGGTTACAAGATTGA
TTGGACATACATTAGCGAAAAAGACATTGATCTGCTGCAGGAAAAAGGTCAACTGTATCTGTTC
CAGATATATAACAAAGATTTTTCGAAAAAATCAACCGGGAATGACAACCTTCACACCATGTACC
TGAAAAATCTTTTCTCAGAAGAAAATCTTAAGGATATCGTCCTGAAACTTAACGGCGAAGCGGA
AATCTTCTTCAGGAAGAGCAGCATAAAGAACCCAATCATTCATAAAAAAGGCTCGATTTTAGTC
AACCGTACCTACGAAGCAGAAGAAAAAGACCAGTTTGGCAACATTCAAATTGTGCGTAAAAAT
ATTCCGGAAAACATTTATCAGGAGCTGTACAAATACTTCAACGATAAAAGCGACAAAGAGCTGT
CTGATGAAGCAGCCAAACTGAAGAATGTAGTGGGACACCACGAGGCAGCGACGAATATAGTCAA

GGACTATCGCTACACGTATGATAAATACTTCCTTCATATGCCTATTACGATCAATTTCAAAGCC
AATAAAACGGGTTTTATTAATGATAGGATCTTACAGTATATCGCTAAAGAAAAAGACTTACAT
GTGATCGGCATTGATCGGGGCGAGCGTAACCTGATCTACGTGTCCGTGATTGATACTTGTGGTA
ATATAGTTGAACAGAAAAGCTTTAACATTGTAAACGGCTACGACTATCAGATAAAACTGAAAC
AACAGGAGGGCGCTAGACAGATTGCGCGGAAAGAATGGAAAGAAATTGGTAAAATTAAAGAGA
TCAAAGAGGGCTACCTGAGCTTAGTAATCCACGAGATCTCTAAAATGGTAATCAAATACAATGC
AATTATAGCGATGGAGGATTTGTCTTATGGTTTTAAAAAAGGGCGCTTTAAGGTCGAACGGCA
AGTTTACCAGAAATTTGAAACCATGCTCATCAATAAACTCAACTATCTGGTATTTAAAGATATT
TCGATTACCGAGAATGGCGGTCTCCTGAAAGGTTATCAGCTGACATACATTCCTGATAAACTTA
AAAACGTGGGTCATCAGTGCGGCTGCATTTTTTATGTGCCTGCTGCATACACGAGCAAAATTGA
TCCGACCACCGGCTTTGTGAATATCTTTAAATTTAAAGACCTGACAGTGGACGCAAAACGTGAA
TTCATTAAAAAATTTGACTCAATTCGTTATGACAGTGAAAAAAATCTGTTCTGCTTTACATTTG
ACTACAATAACTTTATTACGCAAAACACGGTCATGAGCAAATCATCGTGGAGTGTGTATACATA
CGGCGTGCGCATCAAACGTCGCTTTGTGAACGGCCGCTTCTCAAACGAAAGTGATACCATTGAC
ATAACCAAAGATATGGAGAAAACGTTGGAAATGACGGACATTAACTGGCGCGATGGCCACGATC
TTCGTCAAGACATTATAGATTATGAAATTGTTCAGCACATATTCGAAATTTTCCGTTTAACAGT
GCAAATGCGTAACTCCTTGTCTGAACTGGAGGACCGTGATTACGATCGTCTCATTTCACCTGTA
CTGAACGAAAATAACATTTTTTATGACAGCGCGAAAGCGGGGGATGCACTTCCTAAGGATGCCG
ATGCAAATGGTGCGTATTGTATTGCATTAAAAGGGTTATATGAAATTAAACAAATTACCGAAA
ATTGGAAAGAAGATGGTAAATTTTCGCGCGATAAACTCAAAATCAGCAATAAAGATTGGTTCG
ACTTTATCCAGAATAAGCGCTATCTCTAA

HsMAD7 1:

ATGAACAACGGTACAAACAATTTCCAGAACTTTATAGGGATCAGCTCACTCCAAAAGACCCTGC
GGAACGCTCTCATTCCTACCGAGACAACTCAGCAATTTATTGTGAAGAACGGTATAATCAAGGA
AGATGAGCTTAGAGGGGAAAACCGCCAGATTCTGAAAGATATCATGGACGATTACTACCGCGGG
TTCATTAGCGAAACCCTGAGTTCCATTGATGATATTGATTGGACCAGCCTTTTCGAGAAAATGG
AGATTCAGCTGAAGAACGGCGATAACAAAGACACCCTCATTAAAGAGCAGACCGAGTATAGAA
AAGCCATACATAAGAAATTTGCAAATGATGACAGATTTAAAAACATGTTTAGCGCAAAACTCA
TCAGCGATATACTTCCCGAGTTCGTGATACATAACAATAATTACAGCGCCAGCGAAAAGGAGGA
GAAGACACAGGTCATCAAACTTTTTAGTAGATTCGCTACGTCCTTTAAAGACTACTTTAAAAAC
CGCGCTAACTGCTTCTCAGCTGACGATATCTCTTCCAGTAGTTGCCATCGGATCGTGAATGACA
ATGCCGAAATTTTCTTTTCAAACGCCCTCGTCTACAGGCGAATAGTAAAATCCCTGAGCAACGA
TGATATTAACAAAATCTCTGGTGACATGAAAGATTCCTTGAAGGAGATGAGTCTGGAGGAGAT
CTACTCTTATGAAAAATACGGCGAGTTTATCACCCAGGAGGGTATAAGCTTTTACAACGACATC
TGTGGTAAGGTGAACAGCTTTATGAATCTGTATTGCCAGAAAAACAAAGAGAATAAGAATTTG
TACAAGTTGCAGAAGCTGCACAAACAGATCCTGTGTATCGCCGATACCAGCTACGAAGTCCCCT
ACAAATTTGAATCTGACGAGGAGGTGTACCAGAGTGTTAACGGGTTTCTTGACAACATCAGTTC
TAAGCACATTGTGGAGCGGCTGCGAAAGATCGGGGACAATTATAACGGTTATAACCTGGATAAA
ATCTACATTGTGTCCAAGTTTTACGAGTCTGTGTCACAGAAAACCTATCGCGACTGGGAGACAA
TTAACACGGCTCTCGAAATCCACTACAACAATATCCTGCCTGGCAATGGCAAGTCCAAGGCAGA
CAAGGTCAAAAAGGCCGTGAAGAATGATCTGCAGAAGAGTATTACTGAGATCAACGAGCTGGT
TAGCAACTACAAGCTGTGTTCAGACGATAATATCAAAGCCGAAACTTACATCCACGAGATTTCA
CACATCCTGAACAATTTTGAGGCACAGGAACTGAAGTATAATCCTGAGATCCACCTGGTAGAGT
CAGAGTTGAAAGCCAGCGAGCTGAAGAATGTGTTGGATGTGATCATGAATGCCTTCCACTGGTG
TTCTGTGTTCATGACAGAAGAACTGGTGGATAAAGATAACAACTTCTATGCCGAACTGGAGGAA
ATCTACGATGAGATTTACCCTGTCATATCCCTCTATAATCTCGTGAGGAACTACGTGACACAAA
AGCCATATTCTACTAAGAAGATCAAGCTGAACTTTGGCATACCTACTCTCGCAGACGGCTGGAG
TAAATCTAAGGAGTACTCCAACAATGCCATAATCCTTATGCGCGATAATCTGTACTACCTGGGC
ATCTTCAATGCGAAAAATAAGCCAGACAAGAAGATCATTGAGGGGAATACCTCCGAGAACAAA

GGCGACTACAAGAAGATGATCTACAATCTGCTCCCAGGTCCAAACAAAATGATCCCTAAGGTGT
TCCTCTCATCAAAAACCGGGGTAGAGACATACAAACCAAGCGCCTACATCCTGGAAGGATACAA
GCAGAACAAGCACATTAAGTCATCCAAAGACTTCGATATAACATTCTGTCATGACCTCATCGAC
TACTTTAAGAATTGCATCGCTATTCACCCCGAATGGAAAAACTTCGGCTTCGATTTTTCCGATA
CCAGTACTTACGAGGATATTAGCGGCTTTTACAGAGAAGTGGAGCTGCAGGGCTACAAAATTGA
TTGGACATACATCAGTGAGAAAGACATCGACCTCCTCCAGGAGAAGGGGCAGCTTTACCTTTTC
CAGATCTATAACAAAGACTTCTCTAAGAAAAGTACCGGGAACGATAACCTGCACACCATGTACC
TGAAGAATCTCTTCTCTGAAGAAAACCTCAAGGACATTGTGCTTAAACTCAATGGGGAGGCCGA
GATCTTCTTTCGAAAAAGCTCAATCAAGAACCCTATTATTCACAAGAAGGGAAGTATCCTCGTC
AATCGCACATACGAGGCCGAGGAGAAGGACCAATTTGGTAACATCCAAATTGTCAGGAAAAACA
TACCAGAGAATATCTATCAGGAACTTTATAAGTACTTTAATGATAAAAGCGACAAGGAGCTTTC
CGACGAGGCGGCTAAATTGAAGAATGTGGTCGGCCATCACGAAGCCGCAACAAACATTGTTAAG
GACTATCGGTACACTTATGACAAATATTTCCTGCACATGCCGATTACTATTAACTTTAAGGCTA
ATAAGACGGGATTTATCAACGACAGGATTCTGCAGTATATAGCCAAAGAGAAAGACCTCCACGT
GATCGGCATCGATCGGGGTGAGAGAAACTTGATCTACGTTTCCGTGATCGACACCTGTGGTAAT
ATCGTGGAGCAAAAATCTTTTAACATTGTGAATGGCTACGACTATCAGATTAAGCTGAAACAGC
AGGAGGGTGCAAGGCAGATCGCCCGAAAGGAGTGGAAGGAAATCGGGAAAATTAAGGAAATCA
AGGAAGGCTATCTGTCCCTGGTCATTCACGAAATAAGTAAAATGGTGATTAAGTATAACGCGAT
AATAGCCATGGAGGACTTGAGTTACGGCTTCAAGAAAGGTCGCTTCAAGGTCGAGAGGCAGGTA
TACCAGAAATTCGAGACAATGTTGATTAACAAACTCAATTATCTTGTCTTCAAGGACATTTCTA
TTACGGAGAATGGCGGCCTGCTGAAAGGATACCAGCTCACATATATCCCCGACAAACTGAAGAA
CGTCGGTCACCAATGCGGCTGCATTTTCTACGTCCCCGCTGCTTACACGTCAAAGATCGATCCCA
CAACGGGCTTTGTCAATATCTTTAAATTCAAGGATTTGACCGTCGATGCCAAGAGAGAGTTCAT
TAAGAAGTTTGACAGCATCCGCTACGACTCCGAGAAGAACCTGTTCTGCTTCACATTCGACTAC
AACAACTTTATTACCCAGAATACTGTTATGTCTAAGAGTTCCTGGTCTGTTTACACTTACGGCG
TACGCATCAAAAGAAGATTCGTGAACGGACGATTCTCCAACGAGTCCGACACTATAGACATCAC
CAAAGATATGGAGAAAACACTCGAAATGACCGATATTAACTGGCGCGATGGCCATGATCTGCGG
CAAGATATTATTGACTACGAAATTGTGCAACACATCTTTGAAATCTTCCGCCTGACCGTACAAA
TGCGTAACTCCTTGTCTGAACTGGAGGACCGTGATTACGATCGTCTCATTTCACCTGTACTGAA
CGAAAATAACATTTTTTATGACAGCGCGAAAGCGGGGGATGCACTTCCTAAGGATGCCGATGCA
AATGGTGCGTATTGTATTGCATTAAAAGGGTTATATGAAATTAAACAAATTACCGAAAATTGG
AAAGAAGATGGTAAATTTTCGCGCGATAAACTCAAAATCAGCAATAAAGATTGGTTCGACTTT
ATCCAGAATAAGCGCTATCTCTAA

HsMAD7 2:

ATGAATAACGGAACTAACAACTTTCAGAACTTCATTGGTATATCTTCTCTCCAGAAGACCTTGC
GAAACGCCCTGATACCCACAGAGACTACGCAGCAATTCATCGTCAAGAACGGCATTATTAAGGA
AGATGAACTTAGGGGTGAGAACCGCCAGATCCTGAAGGACATAATGGATGACTACTACAGAGGC
TTCATATCTGAAACATTGAGCAGCATCGATGACATAGACTGGACCTCTCTTTTCGAGAAGATGG
AGATCCAGCTGAAGAACGGCGATAATAAGGATACACTGATTAAGGAACAAACGGAGTATAGGA
AAGCAATACACAAGAAGTTTGCTAATGACGACCGCTTCAAGAACATGTTTTCTGCGAAACTGAT
CAGCGACATACTGCCAGAGTTCGTCATTCATAACAACAATTATAGCGCTTCCGAGAAGGAGGAG
AAAACACAAGTCATTAAATTGTTCTCTCGCTTCGCAACATCATTTAAGGACTATTTCAAGAACA
GAGCTAACTGCTTCAGCGCCGATGATATTTCCTCAAGCAGCTGTCATAGGATCGTGAACGATAA
TGCCGAGATCTTCTTCTCTAATGCTCTGGTGTACAGGAGAATCGTAAAGTCATTGAGCAACGAC
GACATCAATAAGATAAGCGGTGATATGAAAGACTCTCTCAAGGAGATGAGCCTCGAGGAGATCT
ATTCCTATGAAAAATATGGAGAGTTTATCACCCAGGAAGGCATTTCCTTTTACAATGACATATG
CGGCAAGGTCAATTCATTCATGAATCTCTACTGTCAAAAGAACAAAGAGAACAAGAATTTGTAC
AAACTCCAAAAGTTGCACAAGCAGATTTTGTGCATTGCAGATACCTCCTACGAGGTTCCCTACA
AGTTCGAGAGCGATGAGGAGGTGTATCAGTCTGTGAACGGGTTTCTTGACAATATCTCTTCTAA
GCACATCGTGGAGCGCCTCCGGAAAATAGGCGATAATTACAACGGTTATAATCTTGACAAAATT

TACATTGTATCCAAGTTCTATGAATCTGTGTCTCAGAAGACTTACAGAGACTGGGAGACGATAA
ATACCGCTCTCGAGATCCACTACAATAACATCTTGCCAGGGAACGGAAAGAGCAAGGCTGATAA
AGTAAAGAAGGCTGTGAAAAATGATCTGCAGAAGTCTATTACGGAAATCAATGAGCTGGTTAG
TAACTATAAGCTGTGCAGTGACGACAACATTAAAGCTGAAACCTACATCCATGAGATAAGCCAC
ATTCTGAACAATTTCGAGGCTCAAGAGCTCAAGTACAATCCAGAGATTCACCTGGTCGAGTCTG
AATTGAAGGCAAGCGAGCTGAAGAATGTGCTCGACGTAATAATGAACGCATTCCATTGGTGCAG
TGTGTTCATGACCGAGGAGCTTGTGGACAAGGATAACAACTTCTACGCCGAACTCGAGGAAATC
TACGACGAGATTTACCCCGTGATTAGTTTGTATAACCTGGTCCGCAACTACGTCACACAAAAAC
CATATAGTACTAAGAAAATCAAGCTGAATTTCGGCATCCCGACACTTGCGGACGGATGGAGCAA
AAGTAAGGAGTACAGCAATAACGCCATCATCCTCATGCGAGACAACCTGTATTACCTTGGAATC
TTCAACGCTAAGAACAAGCCAGACAAGAAAATCATCGAGGGAAACACCAGCGAAAATAAAGGA
GATTACAAGAAAATGATCTACAATCTCTTGCCTGGACCCAATAAAATGATCCCAAAAGTATTCC
TCTCCAGCAAGACAGGCGTGGAGACTTACAAGCCGAGTGCATATATCCTCGAGGGGTATAAACA
GAACAAGCACATCAAGTCTAGCAAAGATTTCGATATCACTTTTTGTCATGATCTCATCGACTAT
TTTAAGAACTGTATCGCCATTCATCCCGAGTGGAAGAATTTCGGCTTTGACTTCAGCGATACGA
GCACGTATGAAGATATTAGTGGATTTTATAGAGAGGTCGAACTTCAGGGGTATAAAATCGACT
GGACTTACATCAGTGAAAAGGATATTGATCTGCTGCAGGAGAAGGGGCAACTCTATCTCTTCCA
AATCTATAACAAGGATTTTTCCAAGAAGTCCACCGGGAATGATAATCTCCATACTATGTATCTC
AAGAACCTGTTCAGCGAAGAGAATCTCAAGGATATTGTCCTGAAGTTGAATGGGGAAGCCGAG
ATATTCTTCAGGAAGAGTAGCATTAAAAACCCTATAATCCACAAGAAGGGAAGCATCCTGGTCA
ACAGAACATACGAAGCTGAAGAGAAGGACCAGTTTGGCAACATTCAAATCGTTAGGAAGAATA
TCCCCGAAAACATATATCAAGAACTGTACAAGTATTTTAATGACAAATCCGACAAGGAATTGTC
CGACGAAGCGGCTAAGCTCAAAAACGTCGTTGGGCACCACGAGGCTGCTACAAATATTGTCAAA
GACTATCGCTACACATACGATAAGTATTTTCTCCACATGCCGATTACCATCAATTTCAAGGCTA
ATAAGACAGGATTCATTAACGACCGAATCCTCCAGTATATAGCCAAGGAAAAGGACCTCCATGT
GATCGGCATTGACAGGGGCGAACGGAATCTGATCTACGTCAGCGTCATAGATACCTGTGGCAAC
ATCGTGGAACAGAAGTCTTTTAACATCGTGAATGGGTACGATTATCAGATAAAGCTGAAGCAGC
AAGAGGGAGCTAGGCAGATAGCACGGAAGGAGTGGAAAGAAATAGGGAAGATTAAAGAGATCA
AGGAAGGCTACTTGTCCCTTGTGATTCATGAAATTTCTAAGATGGTGATAAAGTACAACGCAAT
CATTGCAATGGAGGACCTGAGCTATGGCTTTAAGAAGGGACGGTTTAAAGTCGAGAGGCAGGTG
TATCAGAAGTTTGAAACCATGCTCATCAACAAGCTGAATTACCTGGTATTTAAAGACATCTCCA
TTACCGAAAACGGGGGACTGCTCAAAGGATATCAGCTTACTTACATCCCCGACAAGCTGAAAAA
CGTGGGCCACCAGTGCGGCTGCATTTTCTATGTGCCTGCAGCCTACACATCTAAAATCGACCCCA
CTACCGGGTTCGTGAACATATTCAAATTTAAAGACTTGACTGTTGACGCTAAGCGGGAGTTCAT
TAAAAAGTTTGATTCCATCCGCTACGATTCCGAGAAGAATCTGTTTTGCTTCACATTCGACTAT
AACAACTTCATCACTCAAAATACGGTGATGTCTAAAAGCAGTTGGAGCGTGTACACATACGGCG
TGCGCATTAAACGCAGGTTTGTCAATGGCCGGTTTAGCAACGAGTCCGATACTATTGACATCAC
AAAAGATATGGAAAAGACTCTGGAGATGACCGATATTAATTGGCGGGATGGACACGATCTGCG
CCAGGACATTATTGATTACGAAATCGTGCAGCACATTTTTGAGATCTTTCGCTTGACGGTACAA
ATGCGTAACTCCTTGTCTGAACTGGAGGACCGTGATTACGATCGTCTCATTTCACCTGTACTGA
ACGAAAATAACATTTTTTATGACAGCGCGAAAGCGGGGGATGCACTTCCTAAGGATGCCGATGC
AAATGGTGCGTATTGTATTGCATTAAAAGGGTTATATGAAATTAAACAAATTACCGAAAATTG
GAAAGAAGATGGTAAATTTTCGCGCGATAAACTCAAAATCAGCAATAAAGATTGGTTCGACTT
TATCCAGAATAAGCGCTATCTCTAA

HsMAD7 3:

ATGAACAATGGAACAAACAACTTCCAAAATTTCATAGGAATATCCTCTCTGCAGAAAACATTGC
GGAATGCTCTCATCCCAACTGAGACAACCCAGCAGTTCATAGTCAAAAACGGGATCATCAAAGA
AGACGAACTGAGAGGCGAGAACCGCCAGATTTTGAAGGACATAATGGATGATTACTATCGGGG
GTTTATTTCCGAGACTTTGAGTAGCATCGACGATATCGACTGGACCTCCTTGTTCGAGAAAATG
GAGATTCAGCTCAAGAATGGGGACAACAAAGATACCCTCATCAAGGAACAGACCGAATACAGGA

AGGCTATCCATAAAAAGTTTGCTAACGACGATCGCTTTAAGAACATGTTTAGTGCCAAACTGAT
TTCTGATATTCTGCCCGAGTTTGTTATTCATAATAACAATTACTCTGCCAGTGAAAAAGAAGAG
AAGACTCAGGTTATTAAGCTGTTTTCTCGGTTCGCGACTAGTTTCAAAGACTATTTTAAGAATA
GGGCTAATTGCTTCAGCGCCGATGACATCTCATCCAGCAGCTGCCATAGGATAGTCAACGACAA
TGCTGAAATTTTCTTCAGCAATGCCTTGGTCTACCGGAGAATCGTCAAGTCCCTGAGTAACGAC
GACATCAATAAAATCTCAGGCGATATGAAGGACTCATTGAAGGAAATGAGTCTCGAAGAAATC
TATTCATACGAGAAATACGGCGAGTTTATCACACAGGAGGGGATTTCATTCTATAATGATATCT
GTGGCAAAGTTAACTCATTCATGAATCTGTATTGCCAGAAGAACAAGGAGAACAAGAACCTGTA
TAAATTGCAGAAGCTGCACAAACAGATTCTGTGTATTGCTGATACCTCTTACGAAGTACCATAT
AAGTTCGAAAGTGATGAAGAGGTCTACCAAAGTGTCAACGGCTTTCTTGACAATATTTCTTCCA
AGCATATTGTCGAGAGGCTGCGGAAGATCGGCGACAATTACAACGGGTACAACCTCGACAAGAT
CTACATTGTTTCTAAATTCTACGAATCAGTCTCCCAAAAGACCTACAGAGACTGGGAAACCATT
AATACCGCCTTGGAGATCCACTACAATAACATTCTCCCAGGGAATGGTAAAAGCAAGGCCGACA
AGGTAAAGAAGGCAGTGAAAAATGATCTTCAGAAATCAATTACGGAAATCAATGAATTGGTGT
CCAACTATAAGCTCTGCAGTGACGACAACATCAAAGCTGAAACCTATATACATGAGATCAGTCA
CATCCTCAACAATTTCGAAGCTCAGGAGCTGAAGTATAACCCAGAGATTCACCTGGTGGAATCT
GAACTTAAGGCGTCCGAACTGAAAAATGTGCTGGATGTGATCATGAACGCCTTCCATTGGTGCA
GTGTGTTCATGACTGAGGAGCTGGTGGATAAAGACAATAATTTCTATGCTGAGCTGGAAGAGA
TCTATGACGAGATCTATCCTGTAATTTCTTTGTACAACCTGGTGCGCAACTACGTTACCCAGAA
GCCTTACAGCACCAAGAAGATCAAGCTTAACTTCGGCATCCCAACCCTGGCTGATGGGTGGAGC
AAATCTAAAGAGTACAGTAACAATGCCATTATTCTTATGCGGGACAATCTGTATTACCTCGGTA
TCTTCAACGCTAAGAATAAACCCGACAAGAAAATCATCGAGGGGAACACTTCCGAAAACAAGGG
GGACTACAAGAAGATGATCTACAATTTGCTCCCAGGACCTAATAAGATGATACCAAAGGTCTTC
CTGAGTAGCAAGACCGGCGTGGAGACGTACAAACCCTCTGCCTATATTCTTGAGGGGTACAAAC
AGAACAAGCACATCAAATCCAGCAAAGACTTTGATATTACATTTTGCCATGACTTGATTGACTA
CTTTAAAAACTGCATTGCTATCCATCCTGAGTGGAAGAATTTCGGTTTTGACTTCTCTGATACA
TCCACGTATGAAGACATAAGTGGTTTCTACCGCGAGGTCGAATTGCAGGGCTATAAGATCGACT
GGACGTATATTTCAGAGAAAGACATCGACCTCCTGCAAGAAAAGGGACAGCTCTACTTGTTTCA
AATCTACAATAAAGACTTCTCAAAGAAATCAACCGGAAATGATAACCTCCACACTATGTATCTC
AAGAACCTCTTCAGCGAGGAAAATCTGAAGGACATTGTCCTCAAACTCAACGGTGAAGCTGAAA
TTTTCTTTCGCAAAAGCAGCATTAAGAACCCGATCATCCACAAGAAAGGCAGTATCCTCGTCAA
CCGAACCTATGAGGCGGAGGAAAAAGACCAGTTCGGTAATATACAGATCGTTCGGAAAAACATC
CCCGAAAATATCTATCAGGAACTGTATAAGTACTTTAATGACAAGTCAGACAAAGAACTCAGTG
ACGAGGCTGCTAAGCTGAAGAACGTTGTGGGCCACCACGAAGCAGCTACCAACATCGTTAAAGA
CTACAGGTACACCTACGATAAGTATTTCCTTCATATGCCAATCACAATAAACTTTAAGGCTAAC
AAGACCGGCTTCATTAACGATCGCATCCTCCAATACATCGCCAAGGAGAAAGACCTGCACGTAA
TCGGTATTGACAGGGGAGAACGAAACCTCATTTATGTGTCTGTCATTGACACTTGCGGAAATAT
CGTCGAACAGAAATCCTTCAATATTGTCAACGGCTACGACTATCAGATCAAGCTCAAACAACAG
GAAGGGGCAAGACAGATCGCTAGAAAAGAGTGGAAAGAGATTGGAAAAATCAAAGAGATTAAG
GAGGGGTACCTGTCTCTCGTTATACACGAAATCAGCAAAATGGTCATAAAATACAACGCAATTA
TAGCCATGGAAGACCTTAGTTATGGTTTCAAAAAGGGTCGGTTTAAGGTTGAAAGGCAGGTAT
ATCAAAAGTTCGAGACTATGCTGATAAATAAGCTCAACTATTTGGTTTTCAAAGATATCAGTAT
CACCGAAAATGGCGGATTGTTGAAAGGGTATCAGTTGACATACATCCCCGATAAGCTCAAGAAC
GTGGGGCACCAGTGCGGCTGTATCTTCTACGTGCCTGCAGCATATACCAGTAAAATTGATCCAA
CGACCGGTTTCGTGAACATCTTCAAGTTCAAAGATCTTACCGTGGACGCTAAGCGGGAATTTAT
CAAAAAGTTCGATTCCATACGATACGACTCCGAGAAAAATTTGTTCTGTTTTACCTTCGATTAT
AACAACTTTATTACCCAGAACACCGTGATGAGCAAATCCTCATGGTCCGTGTACACTTATGGCG
TCCGCATCAAACGACGCTTCGTGAATGGGCGCTTCAGTAATGAAAGCGACACCATTGACATTAC
CAAAGATATGGAAAAGACTCTGGAAATGACCGACATTAACTGGCGAGATGGGCATGATTTGCG
ACAAGATATCATCGACTATGAGATAGTACAGCATATTTTCGAGATTTTCAGACTCACCGTCCAA
ATGCGTAACTCCTTGTCTGAACTGGAGGACCGTGATTACGATCGTCTCATTTCACCTGTACTGA
ACGAAAATAACATTTTTTATGACAGCGCGAAAGCGGGGGATGCACTTCCTAAGGATGCCGATGC

AAATGGTGCGTATTGTATTGCATTAAAAGGGTTATATGAAATTAAACAAATTACCGAAAATTG
GAAAGAAGATGGTAAATTTTCGCGCGATAAACTCAAAATCAGCAATAAAGATTGGTTCGACTT
TATCCAGAATAAGCGCTATCTCTAA

HsMAD7 4:

ATGAACAACGGCACTAACAATTTTCAGAACTTTATCGGAATATCCTCCCTGCAGAAAACCCTTA
GAAATGCTTTGATCCCCACAGAAACCACCCAGCAGTTCATAGTGAAGAACGGGATCATAAAGGA
GGATGAGCTGAGAGGAGAAAACCGGCAGATACTCAAGGACATCATGGATGATTACTACAGAGG
CTTCATTTCTGAGACCCTGAGCAGTATAGATGATATTGACTGGACATCCCTTTTTGAAAAGATG
GAGATCCAGTTGAAAAATGGCGATAACAAGGACACCCTCATAAAGGAGCAGACAGAGTACCGA
AAAGCCATCCACAAAAAGTTCGCTAACGACGACCGGTTTAAAAACATGTTCTCTGCTAAGCTTA
TCTCTGATATCCTCCCCGAGTTTGTCATCCACAACAACAATTATAGCGCTAGTGAAAAGGAGGA
GAAGACACAGGTTATCAAGCTGTTCAGTAGATTTGCAACATCTTTCAAAGACTACTTCAAAAAT
CGGGCCAACTGCTTTTCAGCTGATGACATCAGTAGCAGTAGTTGTCATCGCATTGTGAATGATA
ACGCCGAGATATTTTTCAGTAATGCTCTGGTTTATCGGAGGATTGTCAAGAGCCTGTCTAACGA
TGATATCAATAAAATCTCAGGCGATATGAAGGACTCTCTCAAAGAGATGAGTCTGGAAGAGAT
CTACTCTTATGAAAAGTACGGAGAGTTTATCACACAGGAGGGTATCTCTTTCTATAATGACATA
TGCGGGAAAGTGAACTCATTCATGAATCTGTACTGCCAGAAGAACAAGGAGAACAAAAATCTTT
ACAAACTTCAGAAATTGCATAAGCAGATCTTGTGCATTGCTGACACGTCATATGAGGTTCCTTA
CAAGTTCGAGTCCGACGAAGAAGTGTACCAGTCTGTCAACGGCTTTTTGGACAACATAAGTAGT
AAGCATATAGTGGAACGGCTGAGGAAAATAGGCGATAACTACAACGGATATAATCTGGATAAA
ATCTACATCGTCAGCAAGTTCTACGAGTCCGTCTCTCAGAAAACCTACCGGGACTGGGAAACTA
TCAATACCGCTCTTGAAATCCATTACAACAACATTCTTCCAGGTAACGGCAAGTCCAAGGCCGA
CAAGGTGAAGAAGGCTGTCAAAAACGATTTGCAGAAGTCAATCACCGAAATTAATGAACTGGT
AAGTAATTACAAGCTCTGTTCAGATGACAATATCAAGGCTGAAACGTACATCCACGAGATCAGC
CACATCTTGAATAACTTCGAGGCTCAGGAACTGAAGTATAATCCAGAAATTCATCTGGTCGAAT
CAGAGTTGAAGGCCTCTGAGCTGAAAAATGTGCTGGATGTAATCATGAACGCTTTCCATTGGTG
CTCCGTCTTTATGACCGAGGAGCTGGTGGATAAAGATAACAATTTCTACGCCGAGTTGGAAGAA
ATTTACGATGAGATTTATCCCGTTATCTCACTTTACAACCTTGTGCGGAATTATGTGACACAGA
AGCCTTATAGCACAAAGAAAATCAAGCTGAACTTCGGGATCCCAACCCTGGCTGACGGCTGGAG
CAAGAGTAAGGAATACTCTAATAACGCAATCATCTTGATGCGCGATAACCTCTACTATCTTGGA
ATCTTTAATGCGAAAAACAAACCAGACAAGAAGATCATCGAAGGAAACACATCAGAGAATAAG
GGAGATTATAAGAAAATGATCTATAATCTGCTGCCTGGTCCGAATAAAATGATCCCCAAGGTGT
TCCTCTCTTCCAAAACCGGTGTAGAAACATACAAACCTTCAGCCTACATCCTCGAGGGCTACAA
ACAGAATAAACACATCAAGAGTTCAAAGGACTTCGATATTACGTTTTGCCATGATCTGATTGAC
TACTTCAAGAATTGTATCGCTATTCATCCAGAATGGAAGAATTTTGGGTTCGACTTTAGTGACA
CAAGCACATACGAAGATATTTCCGGCTTCTATCGCGAAGTGGAACTCCAGGGATATAAGATCGA
TTGGACCTACATTAGTGAGAAGGACATCGACCTGCTGCAGGAGAAAGGGCAGTTGTACCTGTTT
CAGATTTACAATAAAGATTTCTCTAAGAAAAGCACCGGGAATGATAACCTGCACACAATGTATC
TGAAGAACTTGTTCTCCGAAGAAAACCTGAAAGATATAGTTCTCAAGCTTAATGGAGAAGCCGA
GATATTTTTCAGAAAGAGCAGCATCAAGAACCCCATCATCCATAAAAAGGGTAGTATTCTCGTA
AATAGAACCTACGAAGCTGAGGAAAAGGATCAATTCGGAAATATCCAAATCGTTAGGAAAAAT
ATCCCGGAGAACATCTACCAGGAGTTGTACAAGTATTTCAACGATAAATCTGATAAGGAGCTGT
CCGATGAAGCTGCCAAGCTCAAGAACGTAGTGGGACACCACGAGGCCGCCACCAACATAGTGAA
GGACTACAGGTACACTTATGACAAATACTTTCTGCATATGCCTATAACAATTAACTTCAAAGCC
AATAAGACCGGCTTCATAAATGATCGCATTCTGCAATATATCGCTAAGGAGAAAGACCTGCACG
TGATCGGGATCGACCGCGGGGAGCGGAATCTCATTTATGTGTCCGTAATAGATACCTGCGGAAA
TATCGTCGAGCAGAAATCATTCAACATCGTGAATGGTTACGACTACCAGATCAAACTTAAACAG
CAGGAGGGTGCCAGACAGATCGCGAGAAAAGAGTGGAAAGAAATTGGAAAGATTAAAGAGATT
AAGGAGGGGTACCTGAGCCTGGTTATTCATGAGATTTCAAAGATGGTGATCAAGTATAACGCCA
TCATCGCAATGGAGGACCTTTCCTACGGGTTTAAAAAGGGGCGGTTCAAAGTGGAACGGCAAGT

TTACCAGAAGTTTGAAACTATGTTGATTAACAAGCTCAACTACTTGGTGTTTAAGGATATTTCT
ATCACAGAGAACGGGGGCCTTTTGAAAGGTTACCAGTTGACATATATTCCAGACAAGCTGAAGA
ACGTGGGCCACCAGTGTGGGTGCATCTTCTATGTGCCAGCTGCCTACACTTCTAAGATTGACCCT
ACAACCGGCTTTGTGAACATCTTTAAGTTTAAAGATCTTACCGTTGACGCCAAGCGCGAATTCA
TCAAAAAGTTCGACTCTATCAGGTATGATTCCGAGAAGAACCTGTTTTGCTTCACTTTCGATTA
CAACAATTTCATTACCCAGAACACAGTCATGTCTAAAAGCTCTTGGAGTGTGTACACCTACGGG
GTTCGCATAAAACGCAGGTTTGTGAACGGACGCTTTTCTAATGAATCCGATACAATCGACATTA
CGAAGGACATGGAGAAAACCCTTGAGATGACAGACATCAACTGGCGGGACGGCATGATCTTAG
ACAGGATATCATCGACTATGAGATTGTGCAGCACATCTTTGAGATTTTCAGACTGACCGTGCAA
ATGCGTAACTCCTTGTCTGAACTGGAGGACCGTGATTACGATCGTCTCATTTCACCTGTACTGA
ACGAAAATAACATTTTTTATGACAGCGCGAAAGCGGGGGATGCACTTCCTAAGGATGCCGATGC
AAATGGTGCGTATTGTATTGCATTAAAAGGGTTATATGAAATTAAACAAATTACCGAAAATTG
GAAAGAAGATGGTAAATTTTCGCGCGATAAACTCAAAATCAGCAATAAAGATTGGTTCGACTT
TATCCAGAATAAGCGCTATCTCTAA

S6: Primers/ssDNA Oligonucleotides

Name	Sequence 5' - 3'
Primer 1	CGTTTCGGAATTCTGTTGTAGTT
Primer 2	CTCGAGACGGGAAGTCTTTAC
OCH1 fwd	ATGGCGAAGGCAGATGGC
OCH1 rev	TTAGTCCTTCCAACTTCCTTCAAATG
PMT2 fwd	ATGACAGGCCGTGTCGACC
PMT2 rev	CTATGGGTCTGATACTCTCCAACTAGGTAG
PMT2 sequencing primer	GATTCAATGCTGCTGCTATTCACTG
TPI1 fwd	ATGGTACGTGCATTGAGTCTCATAAG
TP1I rev	CTAGTTTCTGGAATTAATAATGTCCACGAATTC
RFP fwd	ATGGTTTCTAAGGGTGAGGAAGTTATC
RFP rev	TTACTTATAAAGCTCGTCCATACCGTAC
GUT1 fwd	ATGGGAAAAGACTATACACCACTAG
GUT1 rev	TTAAGCAGTGTCCTTAAGCCAG
Sh blefwd	ATGGCTAAACTCACCTCTGCTGTTC
Sh blerev	TTAGTCCTGCTCTTCTGCGACGAAATG
gRNA *GUT1*	GTCGGAATTTCTACTCTTGTAGATCAACGGTATTGTGTGGAACGAGG CCGGCATGGTCCC
gRNA TTTA 1	GTCGGAATTTCTACTCTTGTAGATGTCACCATGGAGAACAAGAACGG CCGGCATGGTCCC
gRNA TTTA 2	GTCGGAATTTCTACTCTTGTAGATCCACTTGACTAACGAAAAATCGG CCGGCATGGTCCC
gRNA TTTG 1	GTCGGAATTTCTACTCTTGTAGATGTCCAAGAAGACAGGAAAGCCGG CCGGCATGGTCCC

gRNA TTTG 2	GTCGGAATTTCTACTCTTGTAGATGTACTATTGACTCTTGGTTGAGG CCGGCATGGTCCC
gRNA TTTC 1	GTCGGAATTTCTACTCTTGTAGATCTCAGCCAGGCTGGGTTGAATGG CCGGCATGGTCCC
gRNA TTTC 2	GTCGGAATTTCTACTCTTGTAGATTGCAACCAAGTTCAGATGGTTGG CCGGCATGGTCCC
gRNA TTTT 1	GTCGGAATTTCTACTCTTGTAGATGACTACCACGGTCAGGAAGTGGG CCGGCATGGTCCC
gRNA TTTT 2	GTCGGAATTTCTACTCTTGTAGATGAAATTCTGGGACGTCGATACGG CCGGCATGGTCCC
gRNA CTTA 1	GTCGGAATTTCTACTCTTGTAGATCCTTACTGATGACGCCCTTGCGGC CGGCATGGTCCC
gRNA CTTA 2	GTCGGAATTTCTACTCTTGTAGATCTGATGACGCCCTTGCTCTTCGGC CGGCATGGTCCC
gRNA CTTG 1	GTCGGAATTTCTACTCTTGTAGATGAAGTTGAGTCCGTTGACAATGG CCGGCATGGTCCC
gRNA CTTG 2	GTCGGAATTTCTACTCTTGTAGATTGTGGTTGTCCAATCTCCACCGGC CGGCATGGTCCC
gRNA CTTC 1	GTCGGAATTTCTACTCTTGTAGATCGAAGGTATTTCCCTGACAGTGG CCGGCATGGTCCC
gRNA CTTC 2	GTCGGAATTTCTACTCTTGTAGATCAGAAATCAGATCTTCCGCAGGG CCGGCATGGTCCC
gRNA CTTT 1	GTCGGAATTTCTACTCTTGTAGATACAACGGTATTGTGTGGAACGGG CCGGCATGGTCCC
gRNA CTTT 2	GTCGGAATTTCTACTCTTGTAGATGGCTGGATGTCTGGGTGACCAGG CCGGCATGGTCCC
gRNA *RFP* 1	GTCGGAATTTCTACTCTTGTAGATAGTACCCTCATATGGTCTTCCGGC CGGCATGGTCCC
gRNA *RFP* 2	GTCGGAATTTCTACTCTTGTAGATAAGTGGGAAAGAGTCATGAACGG CCGGCATGGTCCC
gRNA Sh ble1	GTCGGAATTTCTACTCTTGTAGATTCCCGTGACTTCGTAGAGGACGG CCGGCATGGTCCC
gRNA Sh ble2	GTCGGAATTTCTACTCTTGTAGATCCGGTGTTGTACGTGACGACGGG CCGGCATGGTCCC
gRNA *TPI1* 1	GTCGGAATTTCTACTCTTGTAGATAAGTTTCCTCCTACGAAAAATGG CCGGCATGGTCCC
gRNA *TPI1* 2	GTCGGAATTTCTACTCTTGTAGATCGTAGGAGGAAACTTCAAAATGG CCGGCATGGTCCC
gRNA *TPI1* 3	GTCGGAATTTCTACTCTTGTAGATTGCGGAGACGTAAACAGTCTTGG CCGGCATGGTCCC
gRNA *TPI* 4	GTCGGAATTTCTACTCTTGTAGATAAGGACTTGGGTGTCCCTTACGG CCGGCATGGTCCC
gRNA *OCH1* 1	GTCGGAATTTCTACTCTTGTAGATTACGGACCCTCACAACAATTAGG CCGGCATGGTCCC
gRNA *OCH1* 2	GTCGGAATTTCTACTCTTGTAGATATTTTCCTTACCGCAGTTACGGGC CGGCATGGTCCC
gRNA *OCH1* 3	GTCGGAATTTCTACTCTTGTAGATTGATACCCGATGATGCAGCATGG CCGGCATGGTCCC
gRNA *PMT2* 1	GTCGGAATTTCTACTCTTGTAGATAGGAAGAACCAGTCACTTCGAGG CCGGCATGGTCCC

gRNA *PMT2* 2	GTCGGAATTTCTACTCTTGTAGATGCTCCTACTATCTCAGACACGGGC CGGCATGGTCCC
gRNA *PMT2* 3	GTCGGAATTTCTACTCTTGTAGATATATCAAGACAACTTGGCTATGG CCGGCATGGTCCC
gRNA *PMT2* 4	GTCGGAATTTCTACTCTTGTAGATAGCCATTTCCACCATTTGCGAGGC CGGCATGGTCCC
gRNA *PMT2* 5	GTCGGAATTTCTACTCTTGTAGATTCGTAAGAAATATGCTGCTCAGG CCGGCATGGTCCC
gRNA *PMT2* 6	GTCGGAATTTCTACTCTTGTAGATGGATCTGGTCTGTTACATTCCGG CCGGCATGGTCCC

S7: gRNAs

**Name**	**Sequence 5' - 3'**
GUT1	CAACGGTATTGTGTGGAACGA
TTTA 1	GTCACCATGGAGAACAAGAAC
TTTA 2	CCACTTGACTAACGAAAAATC
TTTT 1	GACTACCACGGTCAGGAAGTG
TTTT 2	GAAATTCTGGGACGTCGATAC
TTTC 1	CTCAGCCAGGCTGGGTTGAAT
TTTC 2	TGCAACCAAGTTCAGATGGTT
TTTG 1	GTCCAAGAAGACAGGAAAGCC
TTTG 2	GTACTATTGACTCTTGGTTGA
CTTA 1	CCTTACTGATGACGCCCTTGC
CTTA 2	CTGATGACGCCCTTGCTCTTC
CTTT 1	ACAACGGTATTGTGTGGAACG
CTTT 2	GGCTGGATGTCTGGGTGACCA
CTTC 1	CGAAGGTATTTCCCTGACAGT
CTTC 2	CAGAAATCAGATCTTCCGCAG
CTTG 1	GAAGTTGAGTCCGTTGACAAT
CTTG 2	TGTGGTTGTCCAATCTCCACC
RFP 1	AGTACCCTCATATGGTCTTCC
RFP 2	AAGTGGGAAAGAGTCATGAAC
Sh ble 1	TCCCGTGACTTCGTAGAGGAC
Sh ble*BLE* 2	CCGGTGTTGTACGTGACGACG
TPI1 1	AAGTTTCCTCCTACGAAAAAT
TPI1 2	CGTAGGAGGAAACTTCAAAAT

TPI1 3	TGCGGAGACGTAAACAGTCTT
TPI1 4	AAGGACTTGGGTGTCCCTTAC
OCH1 1	TACGGACCCTCACAACAATTA
OCH1 2	ATTTTCCTTACCGCAGTTACG
OCH1 3	TGATACCCGATGATGCAGCAT
PMT2 1	AGGAAGAACCAGTCACTTCGA
PMT2 2	GCTCCTACTATCTCAGACACG
PMT2 3	ATATCAAGACAACTTGGCTAT
PMT2 4	AGCCATTTCCACCATTTGCGA
PMT2 5	TCGTAAGAAATATGCTGCTCA
PMT2 6	GGATCTGGTCTGTTACATTCC
